# Inhibition of Steel Corrosion and Alkaline Zinc Oxide Dissolution by Dicarboxylate Bola-Amphiphiles: Self-Assembly Supersedes Host-Guest Conception

**DOI:** 10.1038/s41598-017-02769-y

**Published:** 2017-06-05

**Authors:** Dirk Schmelter, Arthur Langry, Andrej Koenig, Patrick Keil, Fabrice Leroux, Horst Hintze-Bruening

**Affiliations:** 1 BASF Coatings GmbH, Glasuritstrasse 1, D-48165 Muenster, Germany; 2 Clermont University Blaise Pascal, Institute of Chemistry of Clermont-Ferrand, UMR-CNRS 6296, BP 80026, F-63171 Aubière, France

## Abstract

For many decorative applications like industrial and architectural paints, prevention of metal substrates from corrosion is a primary function of organic coatings. Triggered release of inhibitor species is generally accepted as a remedy for starting corrosion in case of coatings damage. A polyurethane based coating, doped with bola-amphiphiles of varying molecular weight but with a common head group motif that stems from ring-opened alkenyl succinic anhydride, enables passivation of the defect and mitigates cathodic delamination, if applied on cold rolled steel. An antagonistic effect results from the intercalation of the bola-amphiphiles into layered double hydroxide Zn_2_Al(OH)_6_ and subsequent incorporation of the hybrid phase into the organic matrix. In particular higher molecular weight bola-amphiphiles get immobilized through alkaline degradation of the layered framework in the basic milieu at the cathode. By means of sediments from colloidal states it is demonstrated that *in-situ* formed zinc oxide encapsulates the hybrid phase, evidenced by impeded dissolution of the ZnO based shell into caustic soda. While inhibition of steel corrosion results from a Donnan barrier layer, impeded zinc oxide dissolution is rooted in zinc catalyzed bola-amphiphile hydrolysis and layered deposition of the crystalline spacer diol hydrogenated bisphenol-A.

## Introduction

**Figure 1:**
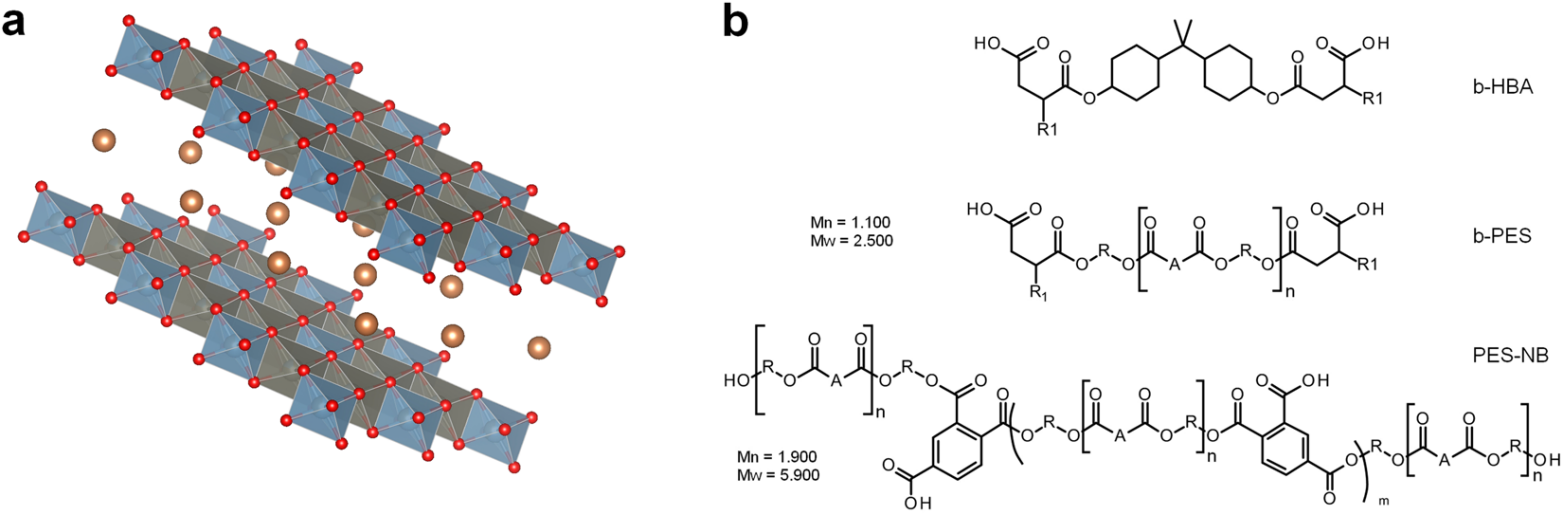
(**a**) Sketch of LDH platelets with intercalated sherical anions (hydrogens are omitted for clarity). (**b**) Formulae of bola-amphiphiles b-HBA and b-PES with octenyl groups “R1” as well as non-bola type polyester PES-NB (cf. methods). For b-PES, “R1” is hydrogenated bisphenol-A (HBA), “A” is an equimolar mixture of dimer fatty and hydrogenated phthalic acid.

Iron, aluminum and their respective alloys are the most widely used metals in civil engineering, infrastructure, transportation and industry^1^. Corrosion of metals is of significant economic relevance and affects numerous industrial applications^2, 3^. Although organic coatings protect the metal against the environment, aggressive electrolytes like chloride may permeate the layer through imperfections^4^ or directly contact the metal surface after coatings failure under mechanical stress, e.g. stone chipping of automotive coatings^5^. Consequently, the design of self-healing coatings has increasingly attracted attention over the recent decade^6^. Contrary to gap-filling via the fracture of capsules filled with curable liquids^7, 8^, or through boehmite crystal growth from calcium aluminate filled coatings under hydrothermal conditions^9^, masking of the substrate surface by a metal-affine compound necessitates less amounts of healing agent and does not require particular external conditions, thus the original character of the coating can largely be kept. Numerous organic molecules like benzotriazole^10^ and 2-mercaptobenzothiazole^11, 12^ as well as oxoanions like molybdate^13^ are well-known, efficient corrosion inhibitors. However, direct incorporation into the paint formulation may harm the barrier property of the coating and may lead to inhibitor loss, e.g. by migration into a top coat layer. Therefore micro- and nano-sized containers have been proposed as hosts for the storage and the use of pH change or ion exchange as trigger for the release of the corrosion inhibitor^14^. Ion exchange of inhibitor anions by electrolyte anions, in particular chloride, appears straightforward. Indeed, in pioneering work, positively charged layered double hydroxides (LDH, cf. Fig. 1) have been tested as a conversion coating on galvanized steel^15^ and with intercalated corrosion inhibitor as a hybrid filler in an organic coating on AA2024-T3 aluminum alloy^16^. Predominantly tested on aluminum alloys, numerous further systems have been described^17^, including direct growth of an LDH surface layer on copper-rich domains^18^, synergistic inhibitor types^19^ and -amino acid interleaved LDH for environmentally friendly inhibition^20^.

However, it has also been demonstrated that LDH-based polymer nano-composites can be applied as a coating layer which provides enhanced stone-chip resistance to an automotive coating system^21^. Evenly dispersed stacks of individually polymer intercalated LDH layers were found either randomly oriented or more homogeneously aligned depending on the formulation. The matrix is interfacially bonded by ionic attraction through carboxylate groups of the polymer. Such reversibly tethered chains are thought to contribute to energy dissipation under mechanical stress in analogy to sacrificial bonding in tessellated bio-composites^22^ and artificial hydrogels^23^.

The work presented here was spurred by the idea to merge the LDH-inhibitor-hybrid concept for corrosion inhibition with the LDH based polymer nano-composite morphology for the prevention of coatings damage, in particular by using carboxylate terminated bola-amphiphiles (BA) as inhibitor species. Generally, BA consist of two hydrophilic head groups attached to a hydrophobic spacer. They are known to self-assemble in aqueous phase into stable lamellar structures, e.g. cell membranes of archaea^24^.

To the best of our knowledge, bola-amphiphiles have not yet been considered for that purpose. Nevertheless, in extension to the “filming amine” theory of corrosion inhibition in the petroleum industry by water- or oil-soluble fatty amines^25^, self-assembling monolayers (SAM) of amphiphilic compounds, applied from solution on the metal oxide or sulfide surfaces have been introduced to promote coatings adhesion and corrosion resistance respectively^26, 27^. Although BA-like compounds have been used, e.g. fatty diamines, only few articles emphasize the BA character of the corrosion inhibitor. Li and Fuhrhop coated gold nano-particles with lipid-like monolayers comprising porphyrin based gaps to study the fixation of solutes in pores at gold electrodes. The assembled, asymmetric bola-amphiphiles, tethered via mercapto groups and terminated with amido groups, protected the gold particles against cyanide dissolution over prolonged time^28^. Diquarternary terminated, epoxy resin based bola-amphiphiles have been shown to inhibit steel corrosion in 2 N HCl solution^29^.

Here we use a recently introduced novel anionic BA motif with different spacers. These are shown in Fig. 1 as b-HBA and b-PES, the latter comprising residual b-HBA, which reflects kinetically controlled step growth polymerization of the ﻿α,β -diol precursor. Their synthesis and assembly in water have been described in detail elsewhere^30^. We point out the peculiar molecular diversity of b-PES (but only 35 b-HBA isomers) and the role of aliphatic chains in α -, resp. β -position to the head groups for the formation of stable assemblies. With R1 = H for instance, b-HBA dissolves in water as bola-electrolyte rather than BA^30^.

In this work coatings are prepared that comprise b-HBA and b-PES in equivalent amounts but disparate states as a result from different processing: direct incorporation from aqueous phase versus prior uptake by LDH via anion exchange in n-propanol. A survey (Fig. S1) maps the general workflow. Although not essential it may serve as center of reference in reading the article.

## Results

### Coatings formulation, film morphology, barrier and mechanical properties

For the coatings formulation, 2-(dimethylamino)ethanol neutralized and water dissolved b-HBA and b-PES were incorporated into the colloidal mixture of film forming polyurethane (PUR), polyester (PES-NB, Fig. 1) and melamine formaldehyde resin. Their amounts were adjusted to match the corresponding BA contents in the LDH based counterparts (LDH-NC), defined by 10 weight-% platelets in the non-volatile fraction and 1:1 charge ratio of BA carboxylate groups to charged aluminum sites. The same was applied for the non-bola type, but amphiphilic polyester PES-NB. We note here, that processing of pristine LDH-Ac for BA intercalation in neat water results in coarse grained hybrid material which persists in LDH-NC. Therefore and for the sake of homogeneous, smooth coatings, the intermediates (LDH-BA) were prepared in n-propanol, thus taking advantage of the “aqueous miscible organic solvent treatment” (AMOST) effect, recently described by M. Yang *et al*.^31^.

**Figure 2:**
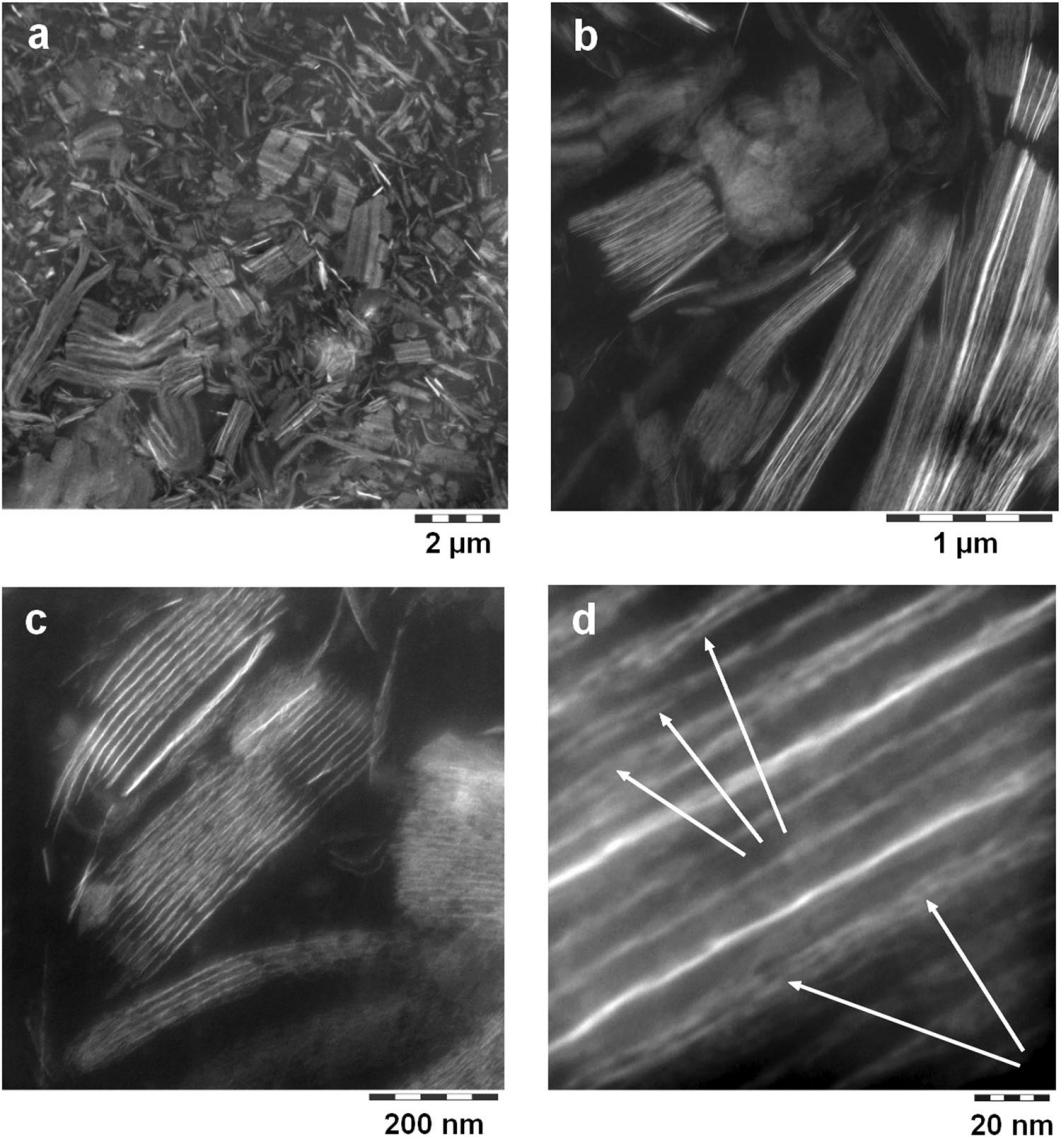
Cryo-TEM pictures zooming into the film of b-PES derived LDH-NC. Arrows in (**d**) indicate fagots of few layers with 2 nm spacing.

**Figure 3:**
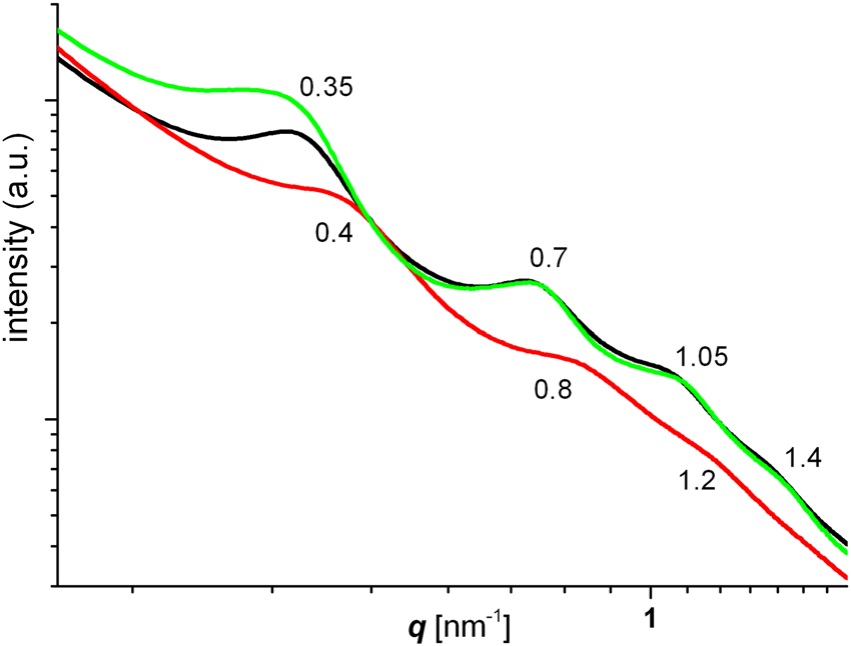
Sections of SAXS curves for LDH-NC films from PES-NB (black), b-HBA (green) and b-PES (red, cf. Fig. 2 for TEM images). Values for the scattering vector *q* indicate hump maxima.

Both, the colloidal LDH-NC and the corresponding films were smooth and transparent (Fig. S2). Films consist of a nanocomposite morphology with evenly dispersed and randomly oriented stacks of LDH platelets (b-PES: Fig. 2, b-HBA: Fig. S3, PES-NB: Fig. S4) that are separated by roughly 15 to 20 nm. Accordingly, small angle scattering (Fig. 3, whole q-range: Fig. S5) shows sets of harmonic humps of lamellar arrays with repeat distances of 15.7 for b-PES and 18 nm for b-HBA and PES-NB. Intriguingly, in case of b-PES, higher TEM magnification reveals a substructure of typically three layers separated by 2 nm (Fig. 2d, arrows). These stay undetected in SAXS around q ~ 2.4 nm^−1^ due to insufficient correlation length while they are barely seen in films obtained from b-HBA and absent in PES-NB derived films.

**Figure 4:**
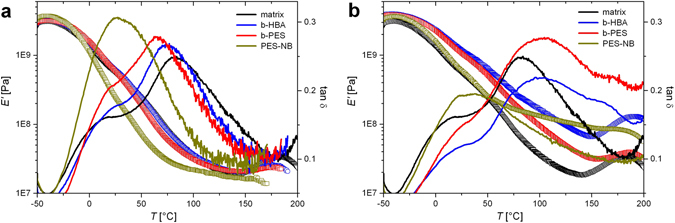
Storage modulus (symbols) and tan *δ* (lines) measured by DMA on (**a**) free films doped with PES-NB, b-HBA and b-PES in amounts matching those in free films of LDH-NC (**b**), both in comparison to a free film of the neat matrix.

Such high amounts of platelets and tethered polymers results in stiffer coatings, as evidenced by thermo-mechanical measurements of free films (Fig. 4b). Remarkably, besides stiffening, both BA based LDH-NC soften the matrix, displayed by a high chain segment mobility above Tg (high tanδ). This contrasts the PES-NB comprising composite with restricted chain movement. Intriguingly, free BA only have a minor impact on the matrix (Fig. 4a). Despite lower storage moduli in the absence of platelets, tanδ values above Tg are distinctly smaller compared to LDH-NC and even the neat matrix. Contrary to the additional amount of PES-NB, which apparently fuses polyester and polyurethane domains, doping the matrix with BA has a minor impact on their tanδ maxima. This counter-intuitive plasticizing of the nano-composites and its absence in the BA doped films implicate reasons for the divergent performance in corrosion inhibition (vide infra) and will be explained on a molecular level with regard to BA self-assembly and anion exchange of acetate versus BA by LDH (cf. chapter “anion exchange”). Finally we note, that high loss moduli (tanδ) may as well reflect residual compounds of low volatility, e.g. 2-(dimethylamino)ethanol and 2-butoxyethanol, as a result of tortuous diffusion paths around platelets and through interstitials. This is demonstrated by control experiments using down to halved film thicknesses of LDH-NC with b-HBA and b-PES (Fig. S6). In both cases the plasticizing above Tg is less pronounced. Overall, the nano-composites provide a poor diffusion barrier for small molecules: only a slightly reduced oxygen permeability compared with the one of the LDH-free counterpart has been measured (Table S1).

### Corrosion inhibition

#### Cathodic delamination on cold rolled steel

The experimental set-up mimics an imperfectly coated (or damaged) object in an aggressive environment. Test panels were coated on one half with LDH-NC, the LDH-free BA doped matrix and the neat matrix for reference respectively. Subsequently the coatings were covered by a non pigmented polyurethane coating in order to assure that inhibitor migration occurs exclusively through the edge of the lower layer. For testing, each uncoated half is exposed to 0.5 M sodium chloride to initiate corrosion and to provide galvanic coupling between the defect and the coated part. Cathodic delamination, one of the fastest and therefore rate determining steps of the corrosion of coated steel, refers to the progressive deterioration of the metal coating interface caused by the cathodic oxygen reduction. Accumulating hydroxyl anions in the confined space raise the pH of the electrolyte, degrade the metal oxide layer and may hydrolyze labile groups of a polymer based coating^32^. Values higher than pH 12 have been reported for coated steel^33^. Using the Scanning Kelvin Probe, the interfacial potential of each specimen was measured over time in the form of successive line scans that spanned the defect and the coated area (Fig. S7). They reveal potential jumps between the active site and the intact substrate-coating interface^34^.

**Figure 5:**
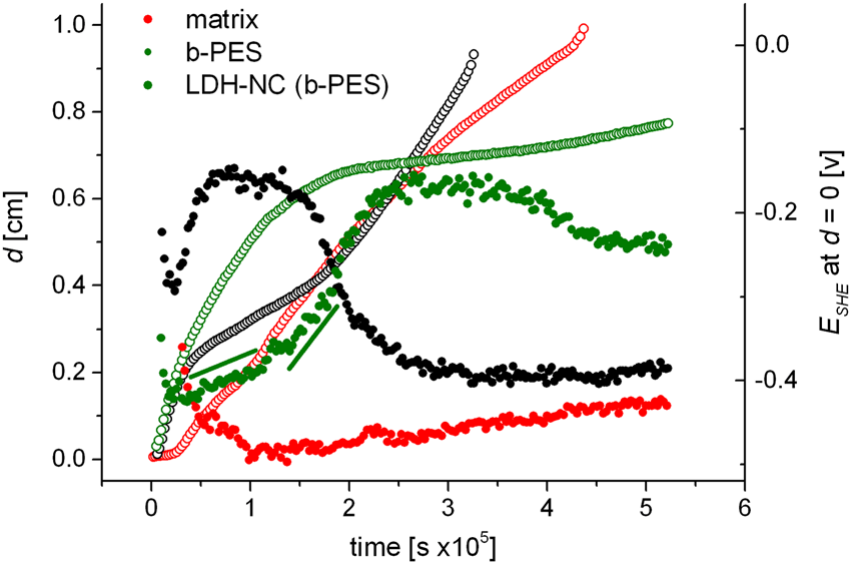
Cathodic delamination of coated steel panels exposed to 0.5 M NaCl. The progress of potential jumps (*d*, outlines) and the evolution of the defect potential (*E*_*SHE*_, solid circles) are superimposed.

The inflection points of the line scans, plotted as a function of time, are shown in Fig. 5 for the matrix, b-PES derived LDH-NC and the LDH-free matrix, doped with equivalent amounts b-PES. They describe the progress of the delamination front and are superimposed on the measured defect potentials. It can clearly be seen that a lower delamination rate coincides with an anodic shift of the defect potential. The matrix itself leads to an intermediate stabilization, while the b-PES doped coating nearly stops the delamination at a later stage, apparently by a diffusion controlled process. However, for the first four and a half days, the data can be fitted with the exponential function *d* = *d*_0_ + *Ae* ^−*αt*^, with α = 1.26 *s* ^−1^ (Fig. S8). The release dynamics depend on the relative concentrations as well as the coefficients for distribution and diffusion of the trigger and the inhibitor in the electrolyte and in the coating. Recent modeling has shown that the special case of a Fickian diffusion determined process with a release exponent α = 0.5 *s* ^−1^ can be reached for a high inhibitor distribution coefficient in conjunction with a high local inhibitor release rate^35^. For low values of the latter which is associated with the dissolution of inhibitor depots, release exponents up to 1.5 *s* ^−1^ may result^35^. Indeed, for a Sr_2_CrO_4_ filled epoxy coating it has recently been shown, that inhibitor diffusion basically takes place through voids, former chromate particles, that are left in an inhibitor depletion zone near the interface with the electrolyte^36^. Compared with the dense network of m-xylylenediamine/2-piperazinoethylamine cured bisphenol-A diglycidyl ether, the organic matrix here is poorly meshed due to low hydroxyl functionalities of the polymers. Nevertheless, molecular dimensions of b-HBA and in particular of b-PES oligomers exceed by far the size of ${{\rm{CrO}}}_{4}^{2-}$ and Cr_2_ ${{\rm{O}}}_{7}^{2-}$ and their diffusivity in the coating is orders of magnitudes lower with regard to hydroxyls (${D}_{OH}\approx {E}^{-5}\,[c{m}^{2}/{s}^{-1}]$)^37–39^ which are thought to trigger release from BA depots (cf. thermo-mechanical properties, preceding chapter) through neutralization of the carboxylic acid head groups (N.B. pristine BA ammonium counter ions leave the film as conjugated base, cf. chapter anion exchange, Fig. S12, Table S2). In accordance with the theoretical model from Tabor and Warszynski^35^ the observed inhibition dynamic suggests disassembly of b-PES depots to be the rate determining process for BA release. However, b-HBA and oligomers from b-PES should migrate with different pace which might be reflected by the two time domains in the anodic potential drift (cf. straight lines in Fig. 5) as well as the delamination curve (arrows in Fig. S8). In that context we emphasize the essential role of the oligomers for inhibition, since b-HBA alone slightly accelerates the delamination rate and barely shifts the defect potential (cf. Fig. S9). Nevertheless, being part of b-PES, b-HBA isomers (of different isomer composition than neat b-HBA) successfully compete with the unidentified, temporarily inhibiting matrix compound for the substrate.

We surmise that shorter bola-amphiphiles, in particular b-HBA, preferentially align normal to the substrate while species of higher molecular weight are homogeneously threaded and provide cohesive strenght through van der Waals attraction. While substrate near head groups attach to metal cations, outward carboxylates are facing the electrolyte and thus build a Donnan barrier for chloride and hydroxyl respectively^40^. The Donnan potential of an ion selective membrane scales with the ratio of the fixed charge density of the membrane over the electrolyte concentration. For the cathodic delamination of a polyester based coating on steel, a potential of 50 to 70 mV was attributed to the sum of pristine carboxylic groups of the polymer and those generated by alkaline hydrolysis of ester groups at pH > 13 in 0.5 M K_2_SO_4_^32^. On the basis of a lipid-like membrane discussed here, the measured anodic shift of the defect potential (200 mV) can thus be attributed to the high charge density of such a layer while the subsequent cathodic drift would be in line with some BA hydrolysis and/or cation exchange through the Donnan barrier.

Strikingly, b-PES based LDH-NC does not show decelerated cathodic delamination over the entire experiment. After a short induction period of 20 minutes, which might reflect the modest barrier against oxygen diffusion (Vide Supra, Table S1), delamination proceeds almost linearly while the defect potential only marginally rises after its initial drop within the first hour. This suggests that the potent inhibitor fraction of b-PES does not reach the metal surface through anion exchange despite excess anions provided by the electrolyte in the detached zone next to the defect due to unimpeded mass transport of chloride from the bulk electrolyte. This contrasts the adjacent, antecedent delamination zone, where, due to the galvanic element, an approximately 3–5 m thick interfacial hydrogel is only accessible for cations (Na^+^) for charge compensation of the hydroxyl anions, the oxygen reduction product^41, 42^. Still, under such alkaline conditions the LDH framework will degrade into ${\rm{Al}}{({\rm{OH}})}_{4}^{-}$ and Zn(OH)_2_, a process that evidently changes the state of BA immobilization, from LDH intercalation probably into Zn^2+^ based ionomers or into encapsulation of the LDH-b-PES hybrid phase (cf. next chapter). In addition, residual acetate from pristine LDH may promote corrosion (cf. chapter “anion exchange”, Table S2).

Finally we note that all coatings equally spread on the slightly rough surface of the substrate (R_*a*_ = 0.5 μm). This indicates that polar Fe_2_O_3_ and FeOOH sites are dominating potentially present, organic carbonaceous species that may resist or are formed during the industrial alkaline degreasing process respectively^43, 44^. Therefore it appears unlikely that the varying performance of the coatings reflects different substrate wetting, the latter conceivably being promoted by some amphiphilic compounds. Imperfect substrate coverage would enhance the probability of ahead potential drops within the zone of the supposedly intact interface. This has been reported for UV cured coatings on iron^45^ but is absent in the present work.

#### LDH degradation & zinc oxide stabilization

**Figure 6:**
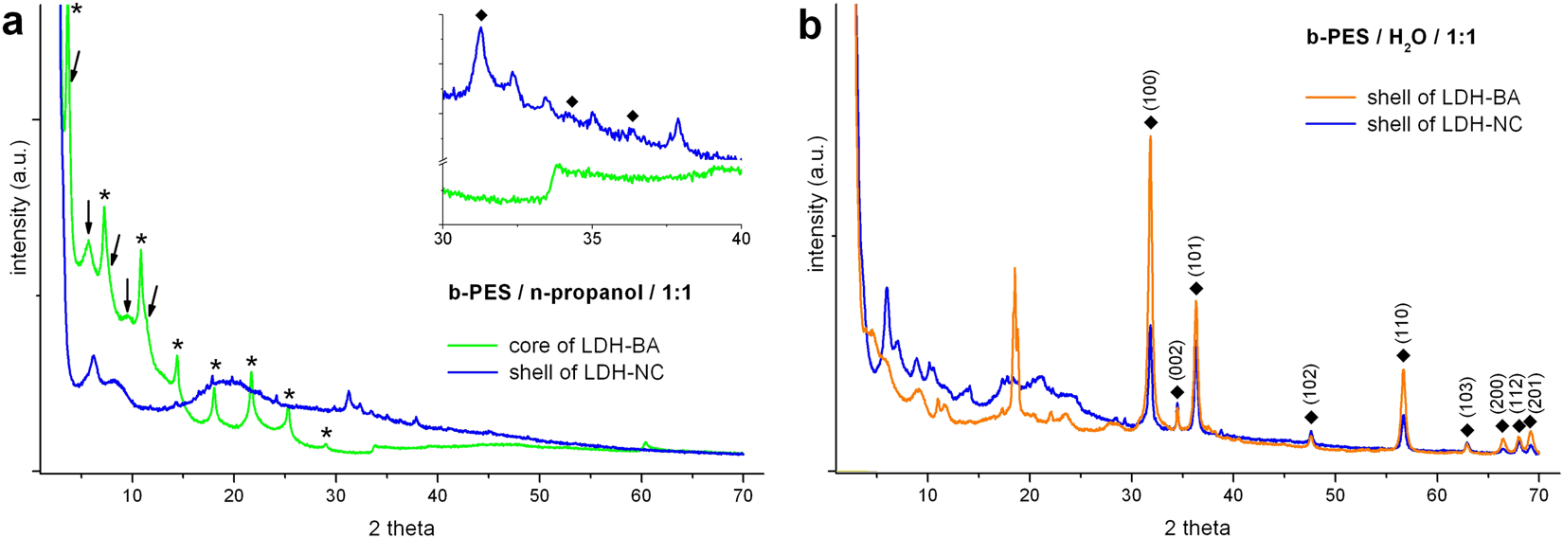
XRD patterns of LDH-BA and LDH-NC sediments after immersion in 10 M NaOH. (**a**) The core of LDH-BA (green) and the shell of LDH-NC (blue), both from n-propanol with equivalent charge ratio. Harmonic peaks caused by two populations of stacked LDH layers with different interlayer spacing are tagged with asterisks (1^*st*^ eight reflexes, d = 2.6 nm) and arrows (2^nd^ to 6^th^ peak, d = 4.7 nm) respectively. The insert shows an enlarged 2*θ* section with those peaks being marked by lozenges that coincide with diffractions on ZnO lattice plains. (**b**) Shells of LDH-BA (orange) and LDH-NC (blue), both from water with an equivalent charge ratio. Peaks that are corresponding to the Wurtzite ZnO structure (cf. JCPDS card no. 36-1451^46^, Table S3) are tagged by lozenges and the corresponding lattice planes.

In the course of analytical tracking of acetate and BA during LDH processing (cf. next chapter, Fig. S1), workup of sediments from colloidal LDH-BA and LDH-NC formulations revealed insolubility of the sediments in concentrated caustic soda (10 M NaOH). However, keeping the same final concentrations, gradual increase of hydroxyl concentration (starting with 2 M NaOH) completely dissolves the precipitates and yields homogeneous solutions. The insoluble counterparts do not visibly change their aspect, neither after shaking over two weeks, nor after sonication or heating to 80 °C. The isolated and water rinsed solids visually consist of a smooth, translucent core and a white, rough shell (cf. Fig. S10). According to XRD, their crystalline constituents can be attributed to the LDH hybrid/nanocomposite and zinc oxide respectively (Fig. 6). Crystallinity of ZnO is low in samples from LDH processing in n-propanol, although the peaks of the Wurtzite lattice are still detectable^46^. Reference samples from processing in neat water yield well crystallized ZnO with a distinctly low intensity of the 002 reflection (2*θ* = 34.5°) which points to anisotropic crystal growth in conjunction with macroscopic sample orientation.

Although basic zinc salt appears as a more straightforward precursor^47^, alkaline induced Zn_2_Al(OH)_6_ transformation into ZnO has also been reported^48^, probably involving zinc hydroxide intermediates^49^, which explains reflections for 2*θ* < 30° (cf. Fig. 6b). The phase diagram of zinc oxide in concentrated sodium hydroxide has recently been refined^50^. The insolubility of zinc oxide in this report is not caused by too high concentrations. For the samples discussed above, calculated contents of 3 weight-% ZnO and 22 weight-% NaOH fall into the solubility regime of the ternary system of ZnO and NaOH with water.

**Figure 7:**
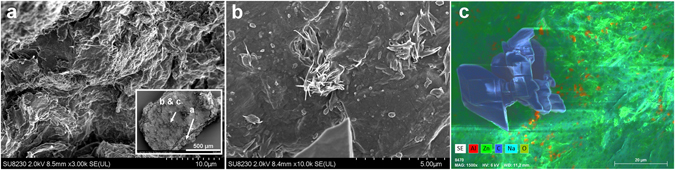
SEM images of a segment (approx. 0.7 × 1.5 mm^2^ lateral size) of the sediment shell from LDH-BA (b-PES in water, cf. Fig. 6b). The locations of images (**a**) to (**c**) are indicated in the insert of (**a**), which shows the top view on the outer shell surface. (**a**) Layered shell structure, displayed by a terrace morphology of the edge. (**b**) A bunch of amorphous material protrudes a diffusion channel. (**c**) Element mapping from EDX discloses the organic nature of crystals found as isolated specks on the outer surface.

Without claiming to unravel mechanistic details, we assume that the distinct stability against dissolution of the LDH hybrid phase, respectively the ZnO based shell initially results from peripheral LDH to ZnO conversion under the action of the LDH as template. In addition BA may direct lateral crystal growth along the interface with the aqueous phase and at the same time the hydrophobic BA layer may protect the oxide from immediate dissolution. Indeed, a side view of the fracture surface of a shell fragment displays undulated edges of a mille-feuille morphology (Fig. 7a). Through interstices this conversion progresses towards the center of the sediment with an ever decreasing rate, the latter being determined by increasingly tortuous diffusion for incoming hydroxyl and leaving ${\rm{Al}}{({\rm{OH}})}_{4}^{-}$. Accordingly, the outer shell surface is speckled with bunches of fibrous material, that protrude from airy structures (Fig. 7b) while only few bunches are found on tighter openings on the crust’s inner side (not shown). Furthermore, they are not congruent with the aluminum enriched specks in EDX mapping (Fig. 7c). Most probably the latter reflect adsorption of Al(OH)_3_ that formed during isolation of the sediment from the alkaline aqueous phase.

**Figure 8:**
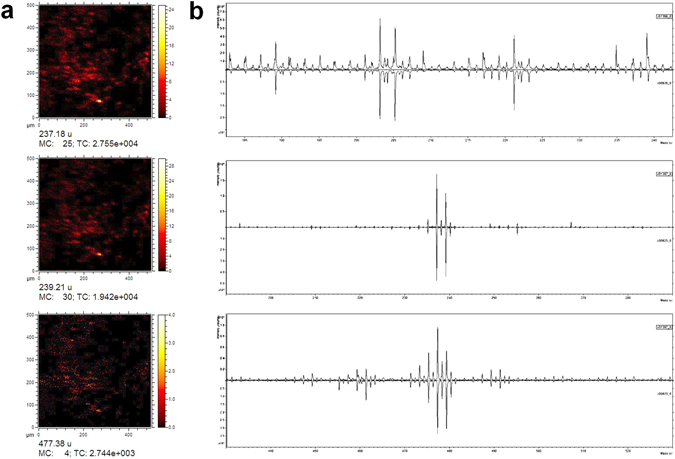
ToF-SIMS, recorded on another fragment of the sediment shell which is shown in Fig. 7. (**a**) Maps (500 * 500 μm^2^) for 237, 239 and 477 mass units (negative polarity, MC = maximum counts per pixel, TC = total counts). (**b**) Selected sections of mass spectra from the sediment shell (up) versus those of the HBA reference (down): top (80–240 u, positive polarity), center (190–290 u, negative polarity), bottom (430–530 u, negative polarity).

Intriguingly, numerous isolated, extended (>20 μm) areas with sleek contours were also found on the outer surface (Fig. 7c). According to EDX these are free of any metal but consist of carbon and oxygen in a ratio close to 7.5, the one of hydrogenated bisphenol-A (HBA). Values of 6.6 and 8.8 were measured with 20 kV and 6 kV respectively. Distinct edges and flat surfaces suggest a crystalline solid which stays unidentified in XRD probably due to limited thickness of oriented layers and a poor contrast. Their chemical nature is corroborated by ToF-SIMS (Fig. 8, Fig. S11). Scans performed on both sides of the crust show domains with sizes comparable to those of the crystals seen by SEM (Fig. 8a, cf. Fig. 7c). The chemical representations in Fig. 8a are based on prominent masses from spectra of high conformity with that of neat HBA (MW = 240.4 g/mol). We note here that SIMS is known to display molecular cluster besides the base peak and fragmentation, the latter becoming increasingly unspecific with lower masses. On the contrary, characteristic peaks for b-HBA and alkenyl succinate (cf. Fig. S11c,d) are basically missing. We surmise substantial hydrolysis of the BA through nucleophilic attack by hydroxyl anions under the catalytic action of zinc. For glycerol phosphate esters a remarkable stability against hydroxyl attack was found even at elevated temperatures whereas hydrolysis readily takes place after conversion of the substrate into electrically neutral derivatives, e.g. metal complexes^51, 52^. In the context of hydrolytic metalloenzymes it has been reported that metallomicelles based on copper as well as zinc complexes boost hydroxyl transfer for substrate hydrolysis^53, 54^. In accordance with Pearson’s “hard and soft acids and bases” concept^55^, catalytic activity of ${\rm{Al}}{({\rm{OH}})}_{4}^{-}$ appears less likely and has not been reported for alkaline ester hydrolysis to the best of our knowledge.

With regard to complete sediment dissolution in a medium with gradually raised hydroxyl concentration, a persistent crust evidently forms in a distinct time-concentration domain which favours ZnO formation, BA hydrolysis and HBA crystallization over LDH degradation and mass flow. We note here that the sediment surface is readily wetted by water and caustic soda despite some surface roughness caused by hydrophobic HBA crystals. Therefore, ZnO stabilization is not due to superhydrophobicity which caused anomalous dispersions of silane treated ZnO nanospikes of hedgehog particles recently described by Bahng *et al*.^56^.

### Anion exchange and BA intercalation

As outlined in the introduction the present work was spurred by the idea to combine the mechanical reinforcement of an LDH-polymer-nanocomposite with the LDH-inhibitor-hybrid functionality by introducing BA as corrosion inhibitor. Anion exchange of BA versus acetate from LDH-Acetate (LDH-Ac) was chosen to circumvent BA loss and purification steps that are usually needed after LDH co-precipitation or *in-situ* synthesis via the polyol route^57–59^. Mass action law entailed, limited degree of BA intercalation was favored over excessive BA which - according to common paradigm - was considered to compromise the coatings barrier function. Here, for the same LDH loading, surplus BA yields brittle films due to less film forming matrix components (not shown). Nevertheless, the amount and the composition of accommodated amphiphiles from molecular diverse BA may not only depend on stoichiometry but as well on the polarity of the solvent. Therefore anion exchange was tracked in n-propanol which was used for LDH-NC formulation (vide supra) in comparison to neat water. The latter was used for the formulation of LDH-free coatings as well as in previous work on BA self-assembly^30^. We note here, that exchanged acetate nearly entirely evaporates as conjugated acid during film formation and baking (Table S2) jointly with the conjugated base of the ammonium counter ions of BA and the film building polymers (qualitative from TGA-FTIR analysis, Fig. S12).

**Figure 9:**
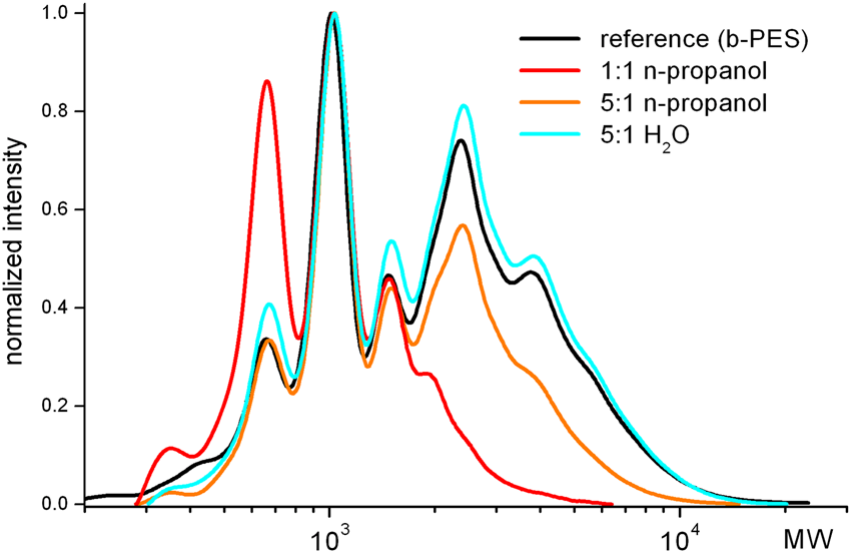
Normalized SEC curves of supernatants obtained from LDH-BA with b-PES (reference, black) in neat water (light blue) and in n-propanol for two charge ratios.

Indeed, as expected, the degree of anion exchange increases with the amount of BA (higher charge ratio) and further added carboxylate groups of the film forming polymers (Table 1). For the same charge ratio and solvent, b-HBA exchanges more acetate than b-PES. Furthermore, with equivalent amounts of BA, the anion exchange is distinctly lower in the organic medium, in particular for b-PES with just 49% of what was found in neat water. Finally, addition of the matrix polymers induces further acetate exchange which is most significant for the b-PES comprising intermediate of the coatings formulation (1:1 charge ratio, n-propanol). These findings are plausible in the light of BA self-assembly in the medium and the extent of ion pair dissociation. Both are less pronounced in n-propanol and thus intercalation suffers from entropic penalty. Weaker mutual attraction of BA enables preferential intercalation of the most hydrophobic species of b-PES: longer chains that are comprising dimer fatty acid moities (Fig. 9).

**Table 1:** Retrieved percentage of acetate from LDH-Ac after: the addition of BA (1^*st*^ number) as a function of stoichiometry (carboxylate to layer charge) and solvent type and after subsequent addition of matrix compounds respectively (2^*nd*^ number).

charge ratio, medium	b-HBA	b-PES
1:1 in H_2_O	80/85	74/90
5:1 in H_2_O	88/91	96/99
1:1 in n-propanol	57/74	36/66
5:1 in n-propanol	88/91	83/85

**Figure 10:**
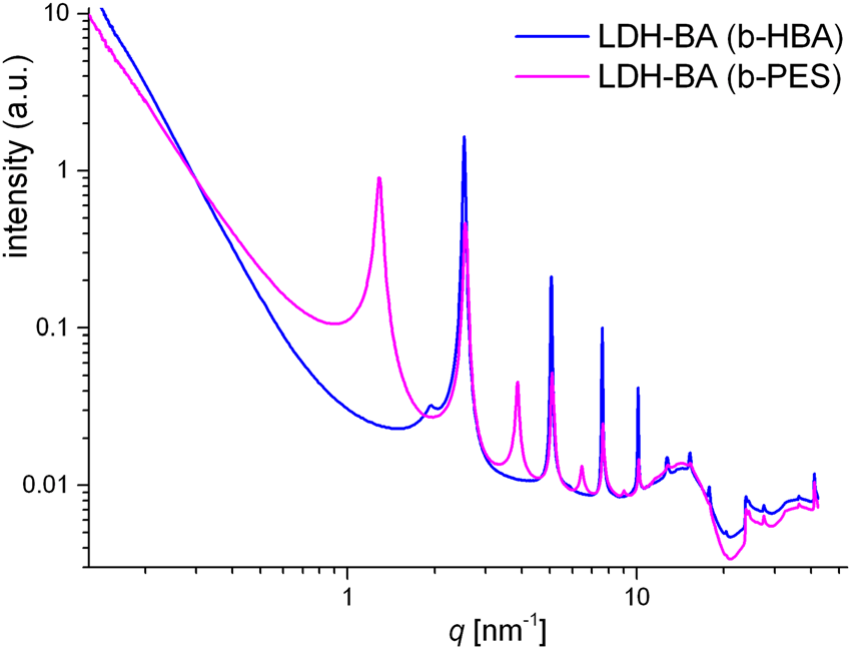
SAXS curves from the intermediate anion exchange of BA versus acetate from LDH-Ac in n-propanol using 1:1 charge ratios (BA carboxylate to layer charge).

Despite moderate BA uptake, both b-HBA and b-PES lead to uniform layer separation with characteristic repeat distances of 2.49 nm and 4.9 nm respectively in LDH-BA, as evidenced by SAXS (Fig. 10) and by cryo-TEM for b-PES comprising LDH-BA (Fig. S13). A slightly smaller repeat distance of approximately 4.0 nm is attributed to the cryo preparation technique. With regard to predominantly much larger spacing in the final coating (cf. Fig. 3), pillaring of LDH by vertically aligned BA appears unlikely. Bilayer accommodation of laterally oriented BA molecules would allow for further polymer uptake and widening of interstices in the second formulation step. Interestingly, for b-HBA, in particular for the cis,trans- and cis,cis-isomers, the separations between carboxylate groups are in good agreement with intralayer Al^3+^ distances. The latter is exemplified by the 100 and 110 lattice plains of the model LDH zincalstibite [Zn_2_Al(OH)_6_][Sb(OH)_6_] with intralayer ordered cations, (Fig. S14)^60^. However, oligomers from b-PES may link platelets through laterally remote Al^3+^ sites. We surmise spatially separated intercalation of different b-PES fractions. Neighboring arrays of several galleries may independently either accommodate BA which suitably adopt a bilayer arrangement or predominantly host sterically demanding BA which are bridging adjacent platelets. The former galleries enable further polymer uptake while the guests of the latter rearrange or collapse during film formation and baking. This scenario, based on partial, fractionated anion exchange, elucidates the LDH stacking in both the colloidal state and the final coating (cf. Fig. 2d).

## Discussion

For corrosion protection through triggered inhibitor release from an organic coating, prevalent paradigm suggests to formulate host-guest inhibitor depots into a polymer matrix in order to maintain the coatings’ barrier function. This work was initiated with a novel approach, namely to align an inhibitor bearing hybrid phase with a layered particle based polymer nanocomposite. The design principle imparts two functions into one morphology: the prevention of coatings damage under stress and the inhibition of subsequent corrosion in case of failure. Stiffening and nanostructuring of the polymer matrix are thought to enhance stress dissipation and the barrier properties of the intact coating. Facilitated release of interstitial inhibitor from distant host layers was expected to counterbalance hampered diffusivity imposed by tortuous paths.

Indeed, intercalation of carboxylate terminated bola-amphiphiles as corrosion inhibitor into layered double hydroxide eventually leads to a nanocomposite morphology with enhanced polymer chain segment mobility in the rubbery state. While that temperature regime is not relevant for corrosion processes under ambient conditions it still indicates plasticizing which can be attributed to free inhibitor. The latter reflects fractionated and incomplete intercalation of largely different species regarding size and hydrophobicity. However, chemical degradation of the host phase in the alkaline milieu near the cathodic site leads to entrapment of at least the essential fraction of the bola-amphiphiles.

On the contrary and in apparent contradiction to prevailing opinion, our results demonstrate that the physical properties of the coating are basically uncompromised by “free” bola-amphiphiles, provided they are incorporated from substantially solvent free aqueous phase. Dispersed in water, the amphiphiles are known to assemble into larger, often anisotropic objects^30^. A consistent image emerges under the assumption, that such entities rather than individual species are accommodated by the matrix and successfully serve as inhibitor depots. The dissolution of the latter - triggered by neutralization of the carboxylic acid head groups - is the rate determining step in inhibitor release according to the cessative kinetics of the cathodic delamination.

With regard to the molecular diversity of the studied bola-amphiphiles, the assembly of anionic species of widely varying molecular weight, driven by van der Waals attraction between hydrophobic backbones as well as terminal side chains is decisive to shield the metal (oxide) surface from corrosive anions through a Donnan barrier. This mode of substrate protection is reminiscent of teichoic acids and other biopolymers that protect halophile and alkaliphile archaea and bacteria in aggressive electrolytes^61^. In the absence of the higher molecular weight fraction, monomeric bola-amphiphile fails to retard the cathodic delamination. We thus discard major contributions of alkenyl succinate to the observed inhibition provided by the molecularly diverse mixture. The conceivable hydrolysis product is a known corrosion inhibitor in concrete as well as on steel electrodes^62, 63^.

Nevertheless, hydrolysis of the bola-amphiphiles gets a boost in the presence of peptized layered double hydroxide. Evidenced by crystalline deposits of the spacer diol (hydrogenated bisphenol-A) and in accordance with the literature we assume hydroxyl transfer on the carbonyl groups of the di(poly)ester, catalyzed by zinc complexes. Zinc oxide formation apparently reflects a template morphology of the pristine composite but may be guided by alkenyl succinate based lamellae, that are thought to form a temporary exterior interface with caustic soda. Concomitantly deposited layers of hydrogenated bisphenol-A pose an obstacle for chemical dissolution of the zinc oxide.

To summarize, we have shown that the assembly of bola-amphiphilic inhibitor compounds into coherent domains may supersede their encapsulation or embedding into an inorganic host. With regard to the latter, chemical instability leads to an antagonistic immobilization of the inhibitor. Found by serendipity, the involved chemical pathway, namely the *in-situ* hydrolysis of (bola-)amphiphiles and the deposition of a hydrophobic crystalline layer, may be considered for the passivation of other metal substrates, e.g. zinc coated steel.

## Methods

### Materials﻿

LDH-Ac has been obtained following known protocols (cf. SI)^21^. XRD curves are given in Figure S15. (Bola)amphiphilic compounds b-HBA and b-PES were synthesized and characterized as described previously^30^. Terminal side chains “R1” are 2-octenyl in this study. Increment “R” of b-PES is 4,4′-isopropylidenedicyclohexanol (hydrogenated bisphenol-A = HBA). Moiety “A” in b-PES stands for an equimolar mixture of dimer fatty acid and 1,2-cyclohexane dicarboxylic acid. PES-NB, has been described previously as PES_h_^21^ with “R” standing for an equimolar mixture of hexane-1,6-diol and 2,2-dimethyl-1,3-propanediol and “A” designating an equimolar mixture of dimer fatty acid and 1,2-cyclohexane dicarboxylic acid. Polyurethane dispersion PUR is a proprietary polymer dispersion (BASF Coatings GmbH, Muenster, Germany) that equals previously described PUR_h_^21^. However, here two PURs are used in a 3:1 ratio of their solid materials, that differ in the type of the diisocyanate. The dominating one is based on 5-isocyanato-1-(isocyanato-methyl)-1,3,3-trimethylyclohexane (IPDI), the other one on 1,3-bis(2-isocyanatopropan-2-yl)benzene. The mean weight molecular weights (Mw) and polydispersity indices (PDI), determined by SEC using PMMA standards, were 50,000 vs. 20,000 and 3.5 vs. 3.8 respectively. Pluriol P900, a polypropylene glycol (BASF SE, Ludwigshafen, Germany) and MF (melamine formaldehyde) resin Resimene HM-2608 (Ineos Melamines GmbH, Frankfurt, Germany) were used as received.

### Coatings Formulations

Prior to addition of b-HBA, b-PES and PES-NB, the LDH-Ac paste (16% solid content in water) was dispersed in n-propanol or deionized water, yielding 3.7 w-% to 8.1 w-% stock dispersions. b-HBA, b-PES and PES-NB were used as ammonium salts from 2-(dimethylamino)ethanol in the form of 60–80 weight-% aqueous dispersions (b-HBA, b-PES), whereas PES-NB is a 60 weight-% solution in a mixture of water and 2-butoxyethanol (1:1 weight ratio). For the preparation of coating materials, a stock solution comprising PUR, PES-NB, MF and P900, the ratios of the non-volatile compounds being 1:0.87:0.82:0.08, was prepared. Different amounts were added under magnetic stirring to LDH-BA, in order to yield 10 weight-% of the LDH framework in the solid proportion of the whole LDH-NC formulation. For the LDH free coatings appropriate amounts of b-HBA, b-PES and PES-NB were added to the matrix stock solution to match corresponding BA contents of LDH-NC. Alcohol from transetherification of the MF resin was not considered in the calculation of non-volatile contents.

### Coatings Application

Formulations were drawn on glass for visual inspection and on untreated polypropylene for free film preparation after baking. Typically dry film thicknesses of 15 ± 3 μm for DMA measurement, permeability measurements and structural analysis were realized if not noted otherwise. Appropriate frames, taking into account varying solid contents of the liquid materials were used to adjust the corresponding wet film thickness. Following a 10 minute flash-off under ambient conditions, films were baked for 20 minutes at 140 °C.

For the SKP experiments, coatings formulations were drawn on alkaline degreased cold-rolled steel panels (R_a_ = 0.5 ± 0.1 μm) to yield 30 ± 3 μm thick dry films. After 30 min flash-off under ambient conditions the films were baked for 30 min at 145 °C. A commercial, solvent borne 2 K PUR clear coat material was subsequently applied as top coat (30 μm dry film thickness). It consists of of a hydroxyl functional poly(meth)acrylate copolymer and a hexamethylene diisocyanate derived isocyanurate. The specimen were baked again for 20 minutes at 140 °C.

### Characterization and Testing

X-ray diffraction patterns (SAXS, WAXS) were recorded on a SAXSess mc^2^ diffractometer (Anton-Paar, Graz, AT) using line collimation and Cu-K radiation.

Small-angle X-ray scattering (SAXS) on LDH-NC films was recorded on the ID02 high brilliance beamline of ESRF, Grenoble, France, using monochromatic radiation (=0.1 nm), a source-sample distance of 55 m and sample-detector distances (max range 1 to 10 m) to cover a typical q-range of 0.0025 to 3.8 nm^−1^, i.e. distances between 2.500 to 1.65 nm. Intensities were corrected for empty mica platelets that were used as sample holder.

Samples for electron microscopy were obtained by freezing a droplet of the dispersion or a section of the solid film in liquid nitrogen, then cutting ultrathin sections of 70–120 nm (cryo-TEM) at −80 °C using a UC6 ultra-microtome equipped with an EM FCS cryo chamber and a diamond knife (Leica Microsystems, Wetzlar, Germany). Samples from free LDH-NC films were prepared using the same microtome procedure. Cryogenic transmission electron microscopy (cryo-TEM) was performed on a Tecnai G2 electron microscope (FEI, Hillsboro, NJ, USA) using 200 kV accelerating voltage.

ToF-SIMS spectra were measured on flaky fragments of a sediment shell and reference samples, fixed on silicone-free support, with an IONTOF TOF.SIMS^*IV*^ spectrometer under static SIMS conditions using ${{\rm{Bi}}}_{1}^{+}$ primary ions accelerated with 25 keV. Areas up to 500 * 500 μm^2^ were scanned 52 times with a pixel size of 2 * 2 μm^2^. Different areas were scanned for positive and negative spectra in order to avoid measured sputtering effects in the second spectrum. Hydrogenated bisphenol-A [CAS 80-04-6], sodium 2-octenyl succinate [from hydrolyzed CAS 26680-54-6] and NaOH neutralized b-HBA were used as reference materials.

For SEM-EDX samples were fixed on conductive carbon tape (PLANO Leit-Tabs, Wetzlar, Germany) and coated with a few nanometers of platinum to enhance the electric conductivity (POLARON SC 7640, Quorum Technologies Ltd, UK). Scanning electron microscopy was performed with a HITACHI SU 8230 (Japan). EDX spectra were recorded with a BRUKER QUANTAX equipped with detectors XFlash 6–30 and FlatQUAD 5060 F (Berlin, Germany).

Scanning Kelvin Probe (SKP) measurements were executed with a height-regulated Kelvin Probe (Wicinski – Wicinski GbR, Surface Scanning Systems, Germany)^34^ in air of >95% relative atmospheric humidity (r.h.) at ambient temperature and using 3 weight-% NaCl as electrolyte in the defect. Interface potentials were correlated with respect to the standard hydrogen electrode (SHE) after calibration against Cu/CuSO_4_^34^.

Methodologies applied for thermomechanical characterization, oxygen permeability, analytical tracking of polymers, acetate and metal cations in the supernatants and sediments of the colloidal states or the baked films are given in the electronic supplementary information. Sketches comprising inorganic lattices were created with the VESTA visualization software (J. Appl. Cryst., 2011, 44, 1272).

## Electronic supplementary material

Supplementary Information
