# Supplementary material for: Inhibition of Steel Corrosion and Alkaline Zinc Oxide Dissolution by Dicarboxylate Bola-Amphiphiles: Self-Assembly Supersedes Host-Guest Conception
Source: Sci Rep. 2017 Jun 5;7:2785. doi: 10.1038/s41598-017-02769-y (PMC5459810; doi:10.1038/s41598-017-02769-y)
Supplement: Supplementary file 1 — Supplementary Information [file 41598_2017_2769_MOESM1_ESM.pdf]

Supplementary Information

Inhibition of Steel Corrosion and Alkaline Zinc Oxide Dissolution by Dicarboxylate Bola-Amphiphiles: Self-Assembly Supersedes Host-Guest Conception

Dirk Schmelter[a], Arthur Langry[b], Andrej Koenig[a], Patrick Keil[a], Fabrice Leroux[b] and Horst Hintze-Bruening[a]*

[1] BASF Coatings GmbH, Glasuritstrasse 1, D-48165 Muenster, Germany
[2] Clermont University Blaise Pascal, Institute of Chemistry of Clermont-Ferrand, UMR-CNRS 6296, BP 80026, F-63171 Aubière, France

* horst.hintze-bruening@basf.com

Thermal Analysis:

TG/DTA analysis of dried samples was conducted using STA 409 c and STA449 c thermal analyzers (Netzsch, Selb, Germany), connected to a Tensor 27 FTIR spectrometer (Bruker, Karlsruhe, Germany) for evolved gas analysis. The transfer line was heated to 200 °C.

For DMA analysis, 5 mm wide strips of free films were punched out and mounted on a DMTA IV unit (Rheometric Scientific, Piscataway, NJ, USA) with a free length of 30 mm. Tests were performed in tensile mode (frequency 1 Hz, amplitude 0.2 %) with heating rates of 3 °C/min (-50 – 0 °C) and 2 °C (0 – 200 °C) respectively.

Metal Contents, Acetate Tracking and Size Exclusion Chromatography:

Metal contents were determined with a Spectroblue ICP-OES spectrometer (Spectro GmbH, Kleve, Germany). If necessary, samples were subjected to microwave digestion with nitric acid, using a speedwave 4 device (Berghof, Eningen, Germany).

For acetate tracing, LDH-BA (and LDH-NC) were centrifuged (6 h @ 16.230 x g) and the supernatants were carefully decanted/pipetted as completely as possible. The sediments were dried in a convection oven at 30 °C until their weight remained constant over 24h and then dissolved in sodium hydroxide solution. The 2 step treatment (starting with 2 M NaOH and dissolving eventual residues with 10 M NaOH) lead to solutions of 2.9 – 18 weight-% sediment in 13 weight-% NaOH), whereas the 1 step method (10 M NaOH, 30.1 weight-%) yielded mixtures with 3 – 23 weight-% of sediment content.

Supernatants and sediments were analyzed for acetate, Zn and Al contents. Additionally, polymer size exclusion chromatography was performed on the supernatants. Control samples of similar composition and known acetate content were used for method validation. For SEC analysis of b-PES, control experiments proved that centrifugation does not lead to fractionation.

Free films were extracted for 8 days with dimethyl sulfoxide (3.6 – 5.9 weight-% film) in sealed vials under magnetic stirring at ambient conditions. After pipetting off the clear solutions (filtration over 6 µm mesh if necessary), sodium hydroxide solution (10 N / 30.1 %, 1 : 100) was added to the extracts. The results were verified against control samples of DMEA-acetate in DMSO-NaOH-mixture.

Preparation of the samples for acetate analysis in a 930 Compact IC Flex modular ion chromatograph (Metrohm, Herisau, Switzerland) depended on the form of the particular samples. Clear samples were diluted with the eluent before measurement, whereas dispersions were extracted with Methylbenzene and the resulting solutions were extracted with acidic eluent.

Capillary electrophoresis for low-content acetate analysis was performed using a P/ACE MDQ system (Beckman Coulter, Indianapolis, IN, USA). Samples were diluted with water and filtrated if precipitation occurred.

Permeability Measurements:

Permeability measurements were carried out on free films with testing areas varying between 1 and 5 cm^2, depending on the perviousness of the respective samples.

Oxygen permeability was analyzed with an Oxtran 2/21 SH OTR testing system (Mocon, Minneapolis, MN, USA) in accordance with ASTM D 3985-02 at 23 °C and 0 % r.h. as well as ASTM F 1927-07 at 23°C and 85 % r.h..

Synthesis and Characterization of LDH-Acetate:

LDH-Ac has been obtained via anion exchange, using an 8-fold excess of sodium acetate over nitrate ions intercalated in the starting phase, the latter prepared straightforwardly by co-precipitation. Experimentally, equal volumes of the salts´ solutions ($Zn(NO_3)_2 \cdot 6H_2O$ and $Al(NO_3)_3 \cdot 9H_2O$ in molar ratio of two were added to an aqueous solution of $NaNO_3$. The addition was performed at constant pH of 9.0 ±0.1 under nitrogen flow for 3 hours, followed by 3 hours of ageing. White slurry was retrieved after 3 cycles of centrifugation and washing. ICP-OES analysis confirms a cation ratio for Zn : Al close to two (+/- 0.05 over different batches) and ion chromatography reveals a typical anion ratio of acetate to nitrate of 1.9. XRD curves are shown in figure S15.

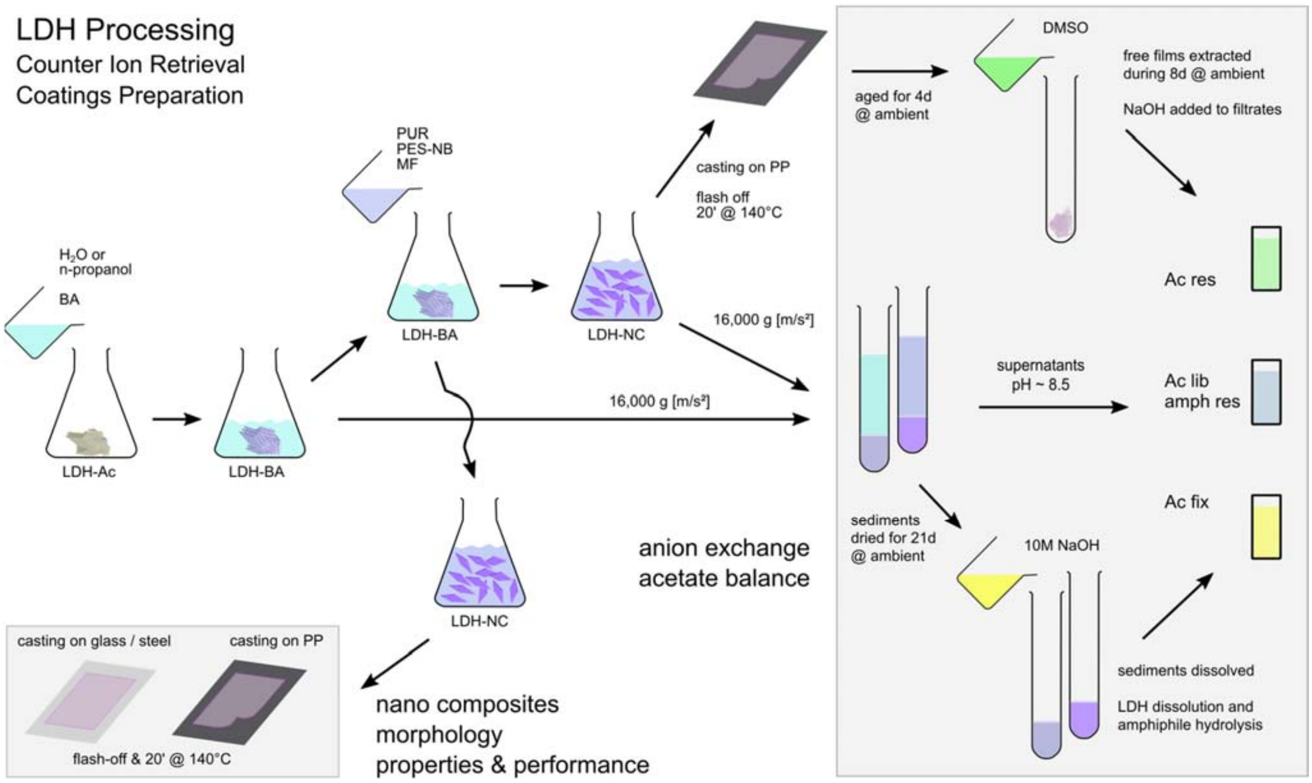

Figure S1. Processing of LDH-Ac for the preparation of coatings formulations (bottom left, above) and for analysis of anion exchange by the bola-amphiphiles b-HBA and b-PES as well as non-bola type amphiphilic polyester PES-NB. LDH-BA and LDH-NC refer to the colloidal states after addition of the amphiphile and the completed coatings formulation respectively. "Ac-res", "Ac-lib", "Ac-fix" and "amph-res" denote residual, liberated and fixed amounts of acetate and amphiphile respectively. For LDH-free coatings the same scheme without the analytical tracking of anion exchange was used. For practical reasons free amphiphiles were added to the recipient containing the matrix mixture.

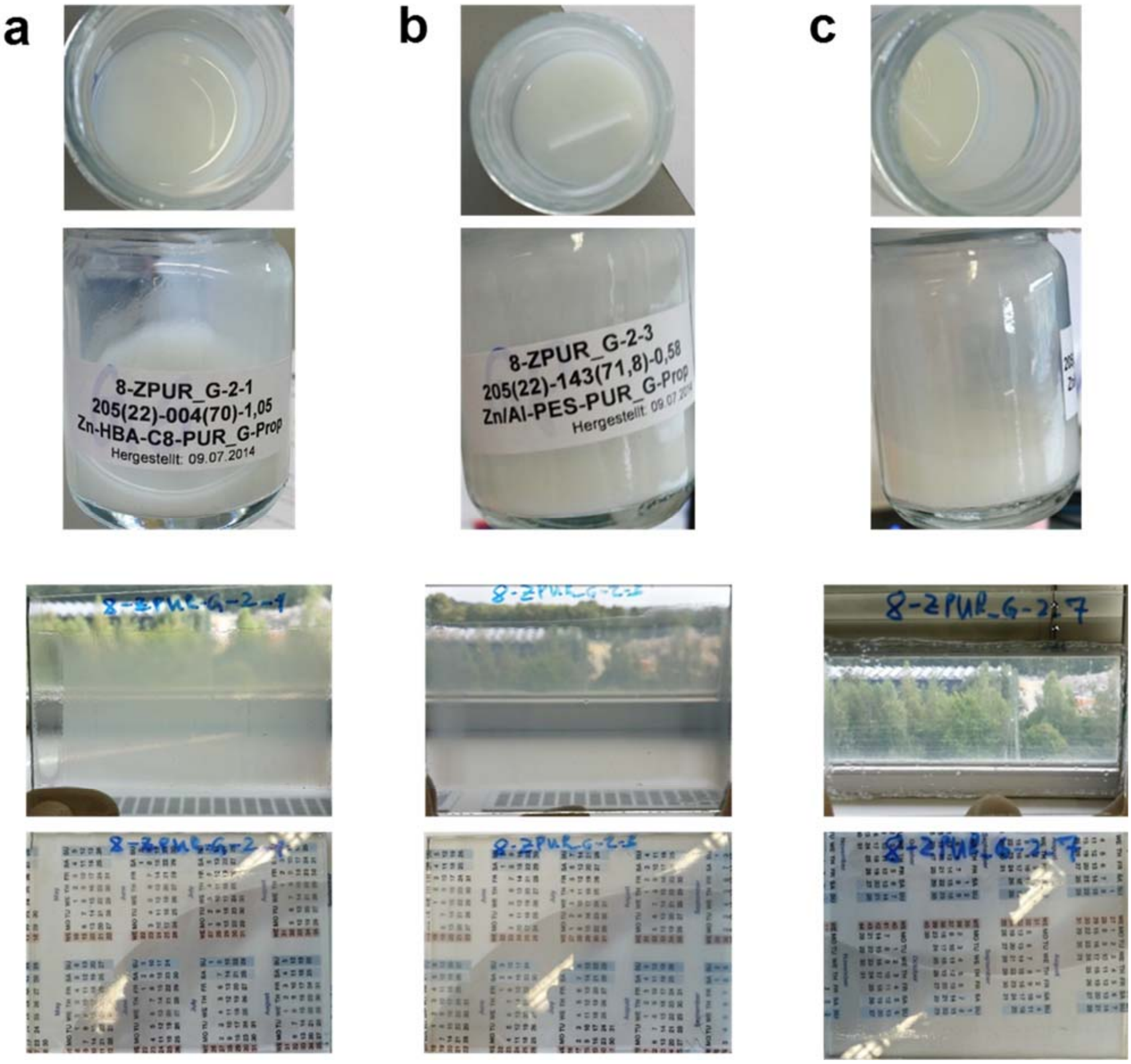

Figure S2. Photographs of LDH-NC dispersions (above) and drawn coatings on glass (bottom) using the very same batches. LDH-NC was prepared from LDH-BA intermediates, that were obtained in n-propanol, using BA in charge equivalent amounts of (a) b-HBA, (b) b-PES and (c) PES-NB. The loading of solid LDH-NC with the LDH framework is 10 weight-% in all coatings.

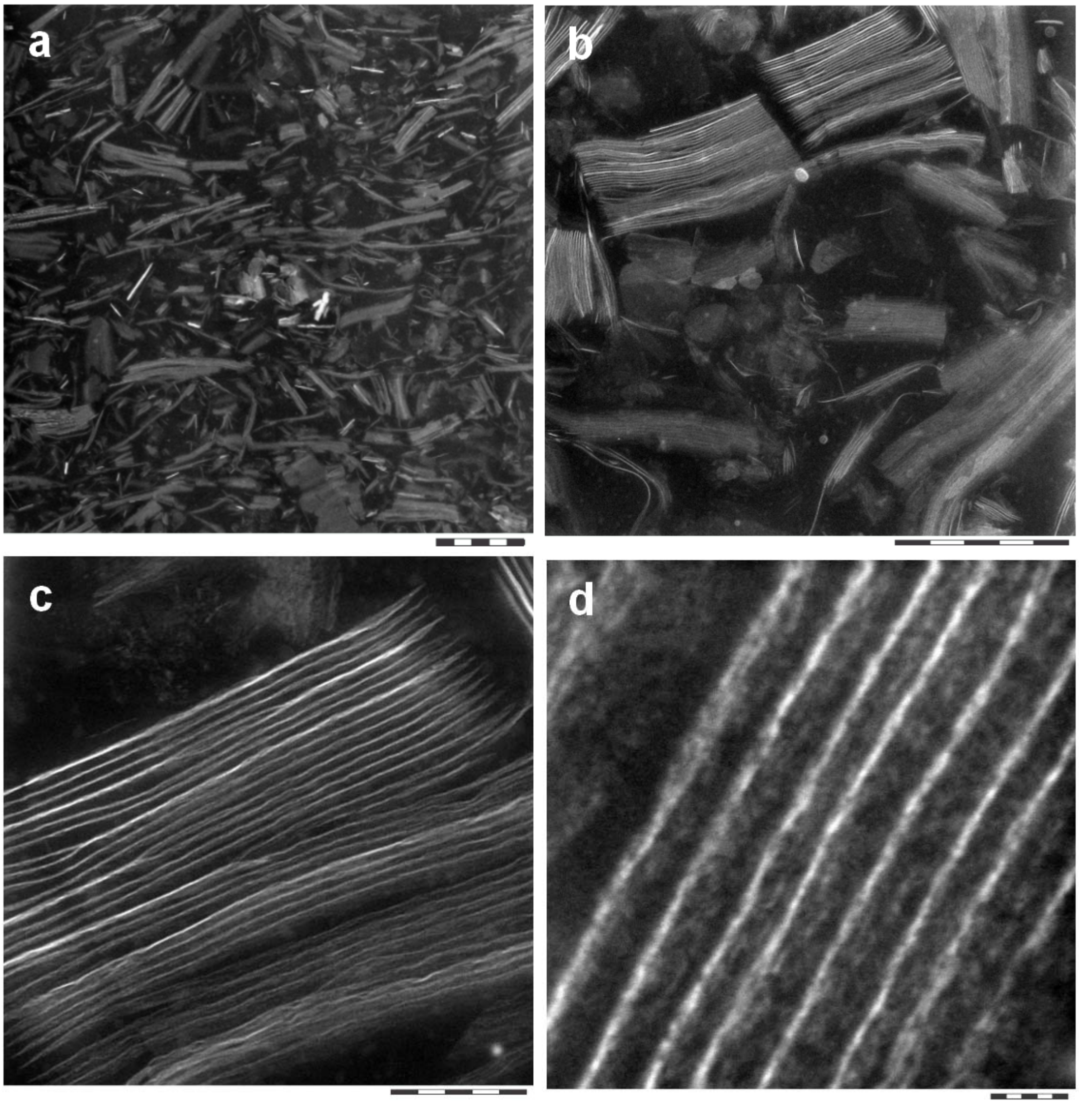

Figure S3. Cryo-TEM pictures zooming into the film of b-HBA derived LDH-NC. Scale bars from (a) to (d) are 2 µm, 1 µm, 200 nm and 20 nm respectively.

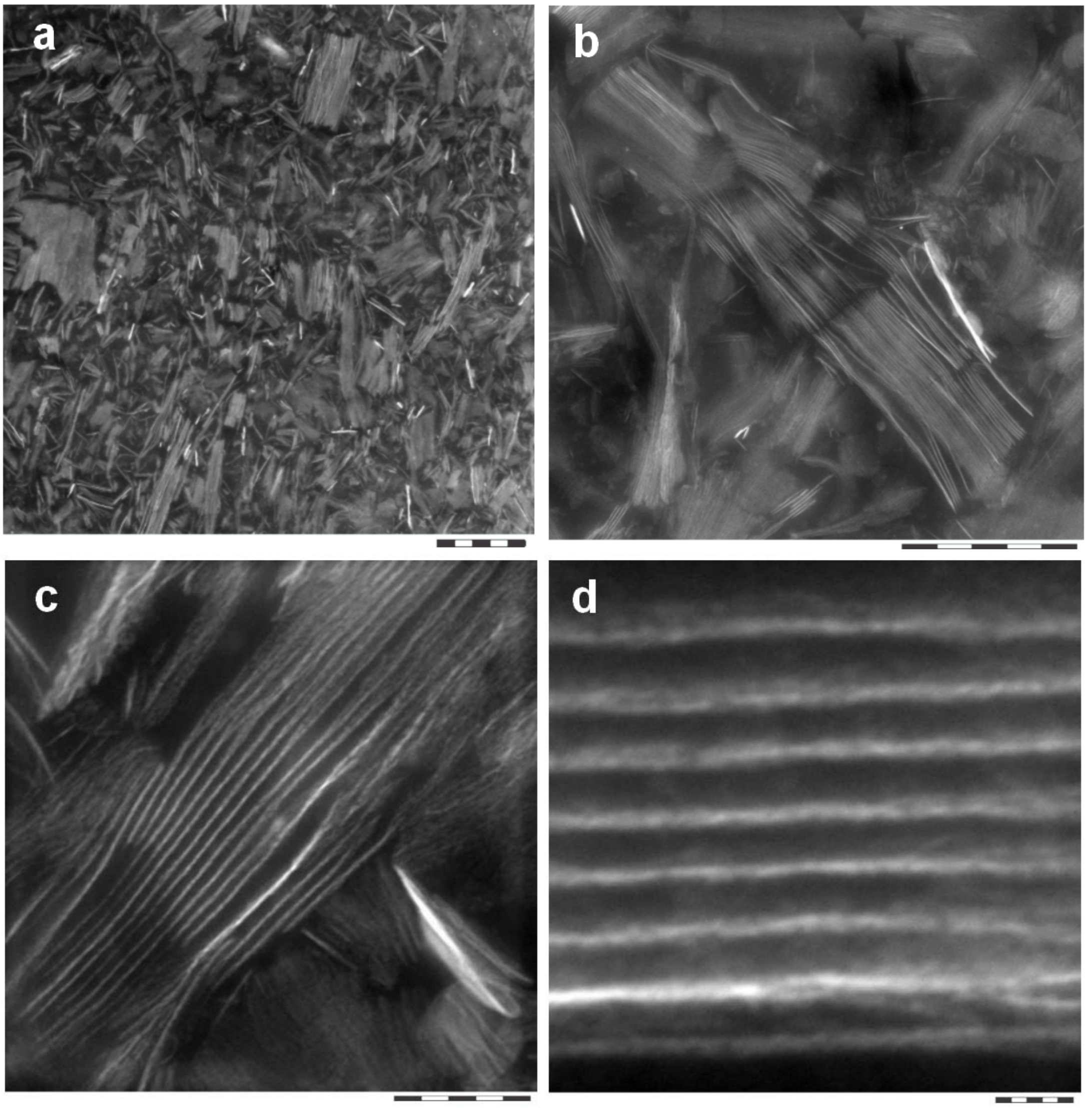

Figure S4. Cryo-TEM pictures zooming into the film of PES-NB derived LDH-NC. Scale bars from (a) to (d) are 2 µm, 1 µm, 200 nm and 20 nm respectively.

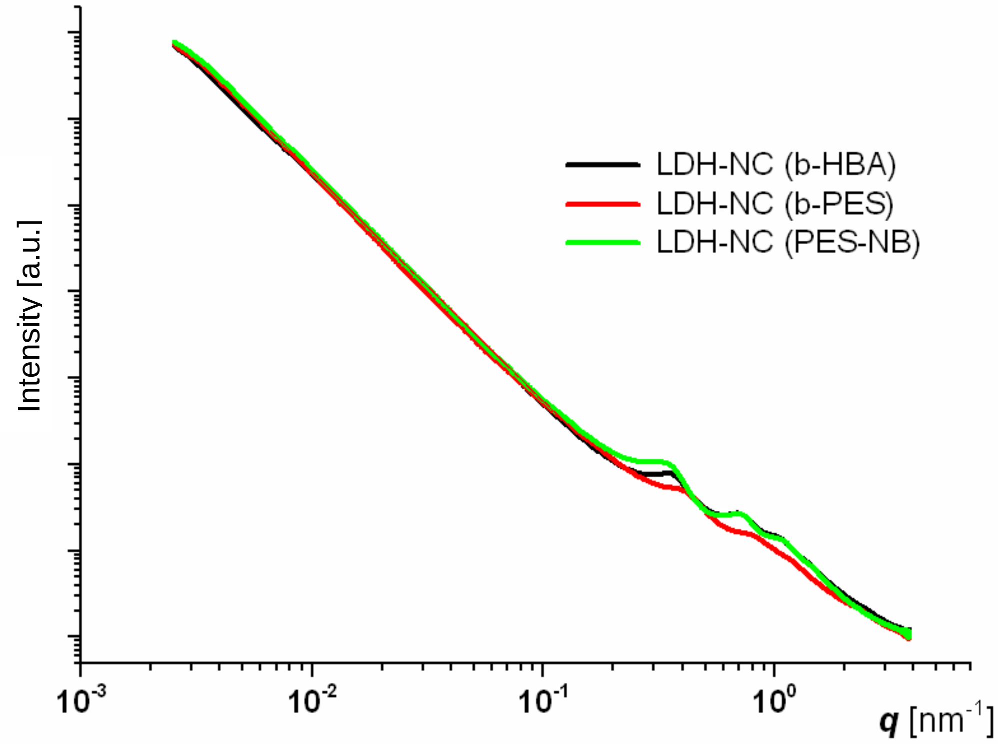

Figure S5. SAXS curves of LDH-NC derived free films. A zoomed section is shown as figure 3 in the article.

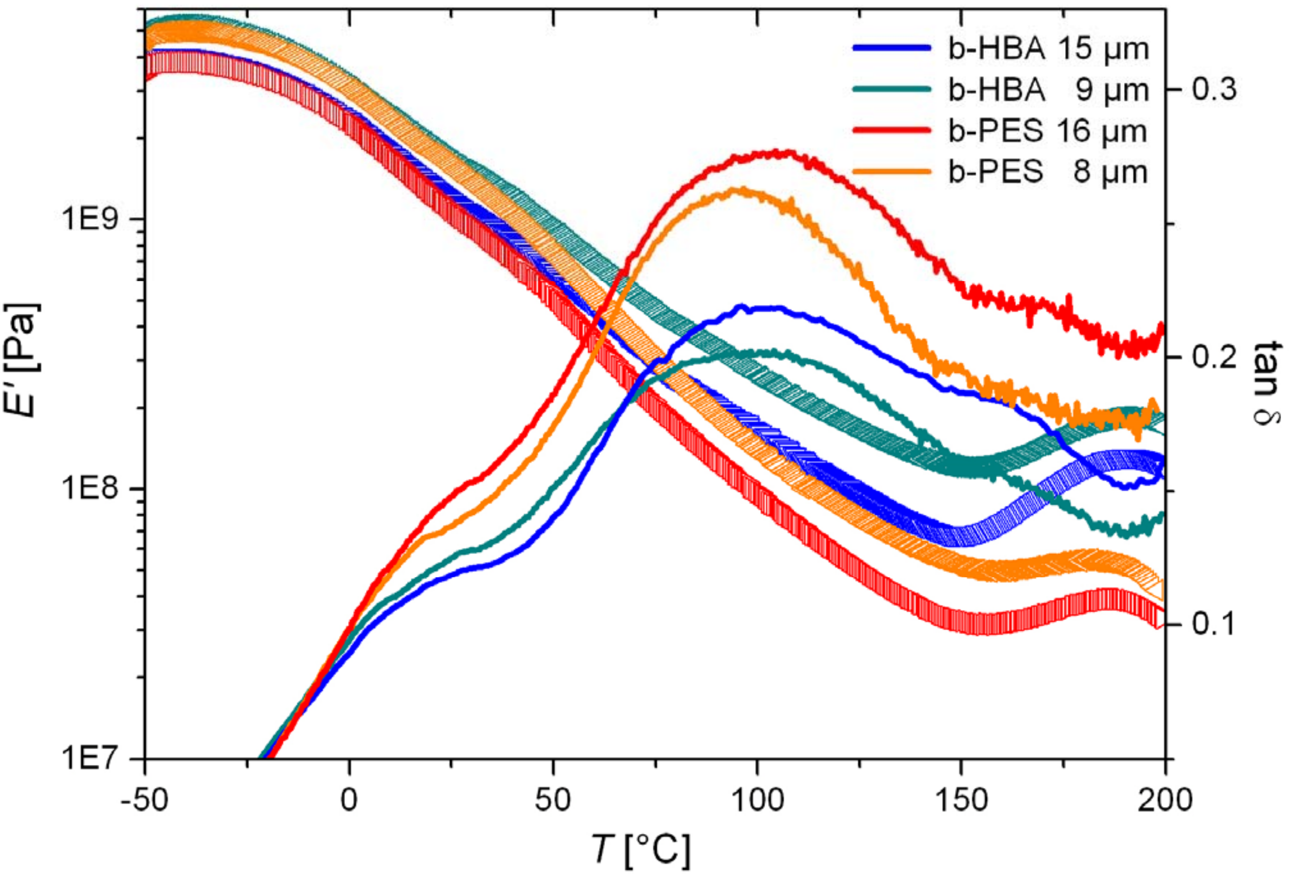

Figure S6. DMA curves obtained from free films, prepared in two different dry film thicknesses from LDH-NC comprising b-PES and b-HBA respectively. Curves for higher film thickness are shown in figure 4b of the article.

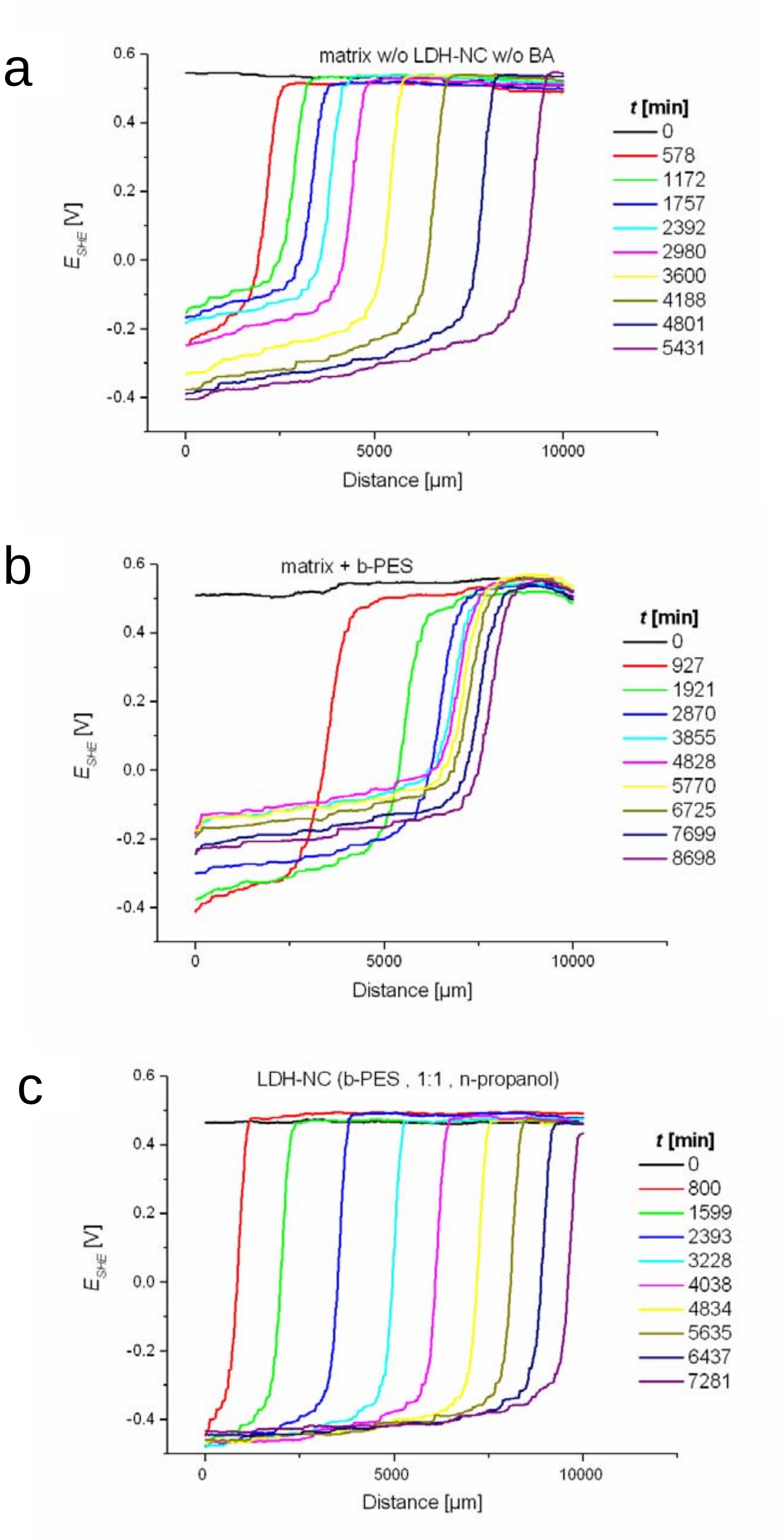

Figure S7. Arbitraryly selected SKP potential lines, measured at different times over the exposure period of 4 to 6 days. The defect is at distance d = 0 µm. Curves for (a) unfilled matrix, (b) matrix doped with b-PES and (c) the LDH-NC nanocomposite obtained from b-PES.

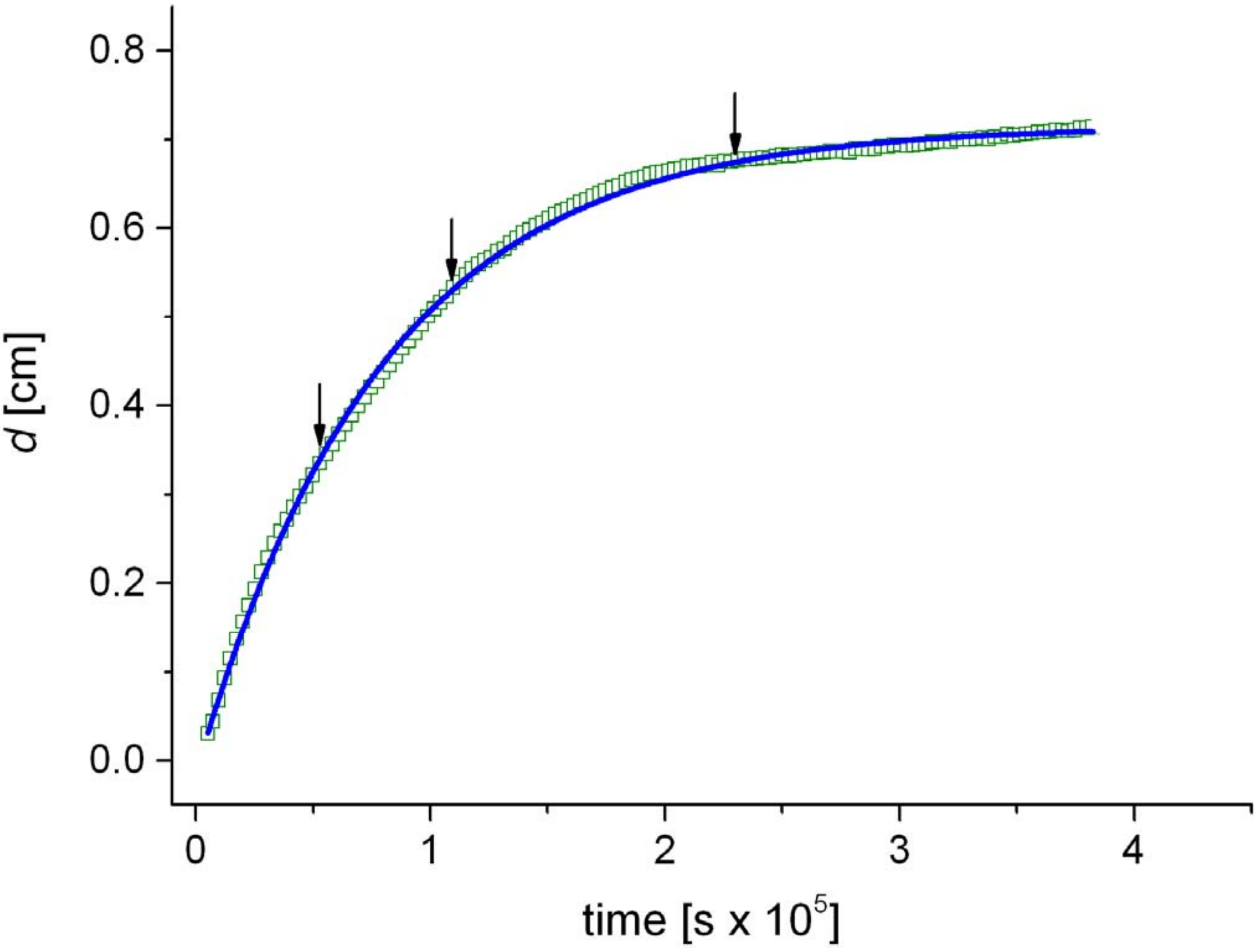

Figure S8. Fitted curve of the cathodic delamination front of b-PES loaded matrix (cf. fig. 5 in article). The fitted curve follows the exponential function $d_t = d_0 + A\ e^{(-\alpha t)}$ with $d_0 = 0.7149$ [cm], A = -0.72918 [cm] , $\alpha = -1.25636$ [s^{-1}] , $X^2 = 3.52\times10^{-5}$. Arrows indicate two time domains seen in the corresponding potential curve (fig. 5 in article).

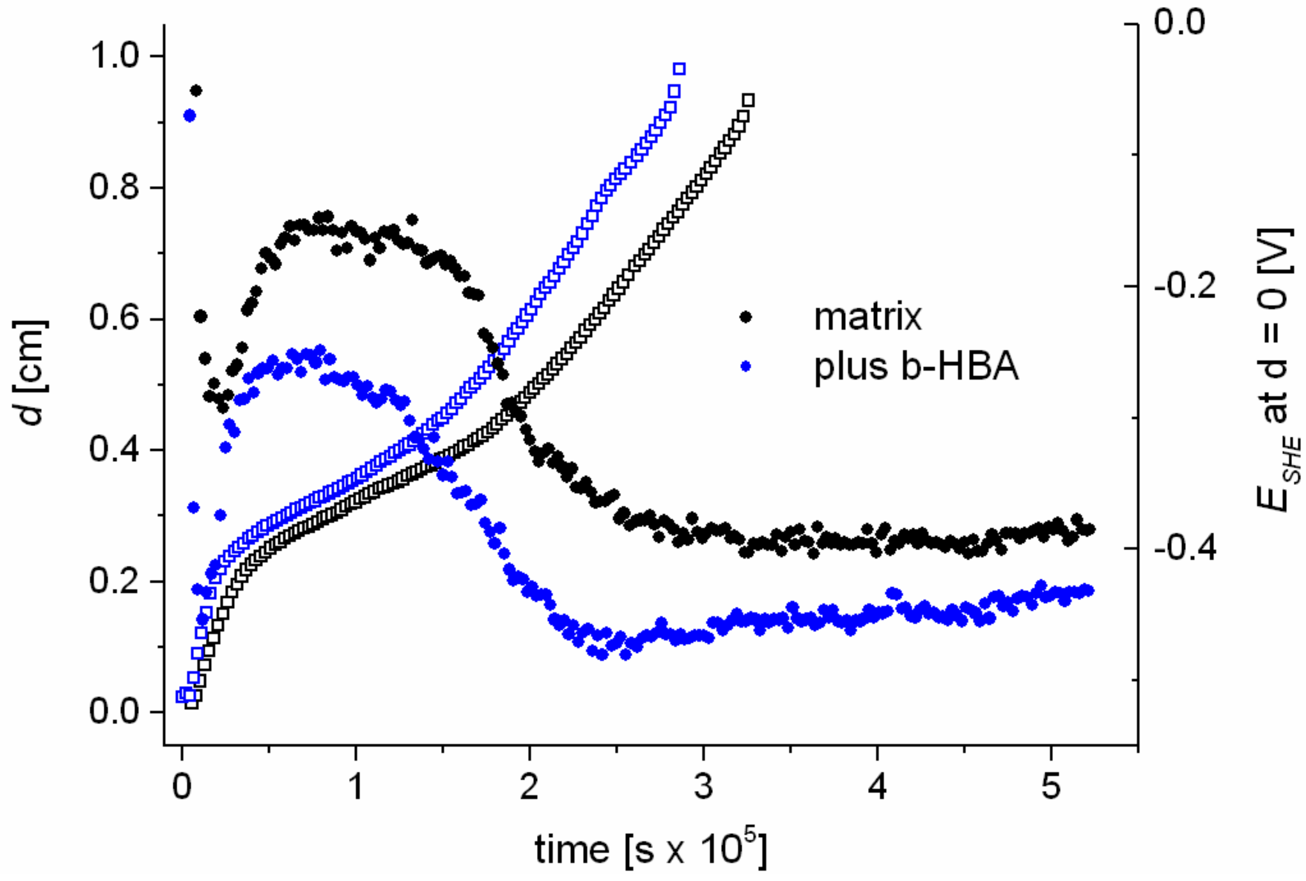

Figure S9. SKP results for cathodic delamination on coated steel for the b-HBA doped matrix and the neat matrix.

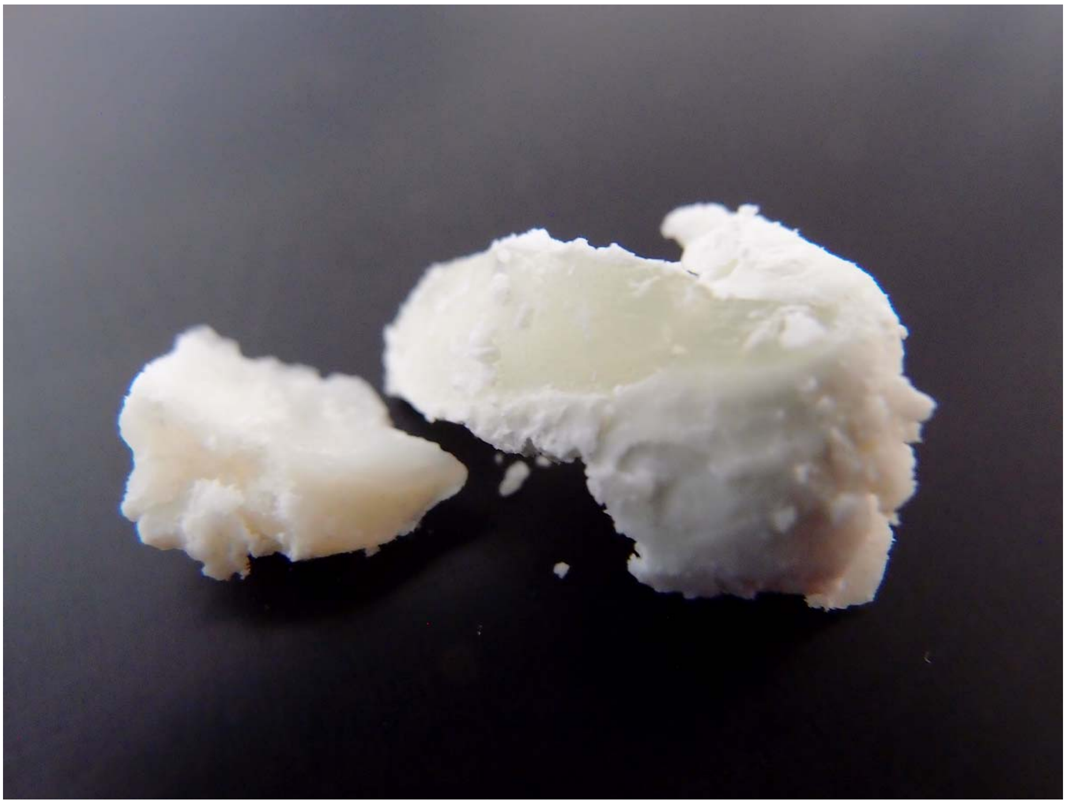

Figure S10. Sediment of LDH-NC dispersion, obtained from b-PES based LDH-BA in n-propanol and charge ratio 1:1, after prolonged treatment with 10 M NaOH. A white shell encapsulates a rubbery, smooth and translucent core.

Figure S11 a. ToF-SIMS spectra of the caustic soda treated LDH-NC sediment shell surface. Peaks of positive polarity are pointing upwards, those of negative polarity downwards. N.B.: real peak shape and fine structure (and thus precise mass) depend on representation resolution.

File: V01397_0.ITS	Date: Thu Feb 09 09:54:17 2017	Polarity: **Positive / Negative**
Sample Info:<br>**Sample:** **24149@ A** **Sediment, Probe wie 24056**<br>Order: 2017 0045 B<br>Origin: **BASF**		Primary Beam:<br>Species: Bi1<br>Area: 500 x 500 µm²<br>Dose: 2.05E+09

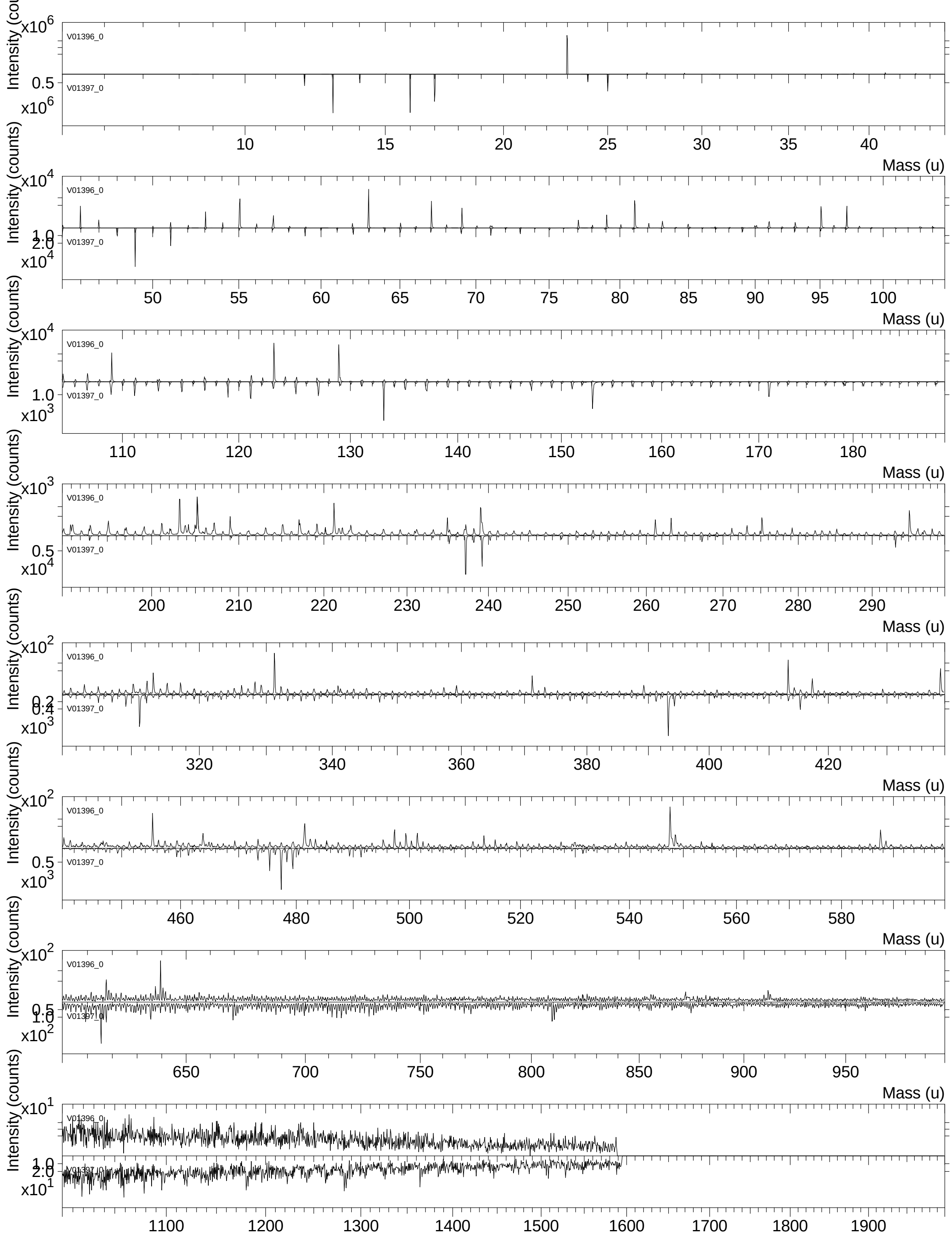

Figure S11 b. ToF-SIMS spectra of hydrogenated bisphenol-A (MW = 240.4 g/mol). Peaks of positive polarity are pointing upwards, those of negative polarity downwards. N.B.: real peak shape and fine structure (and thus precise mass) depend on representation resolution.

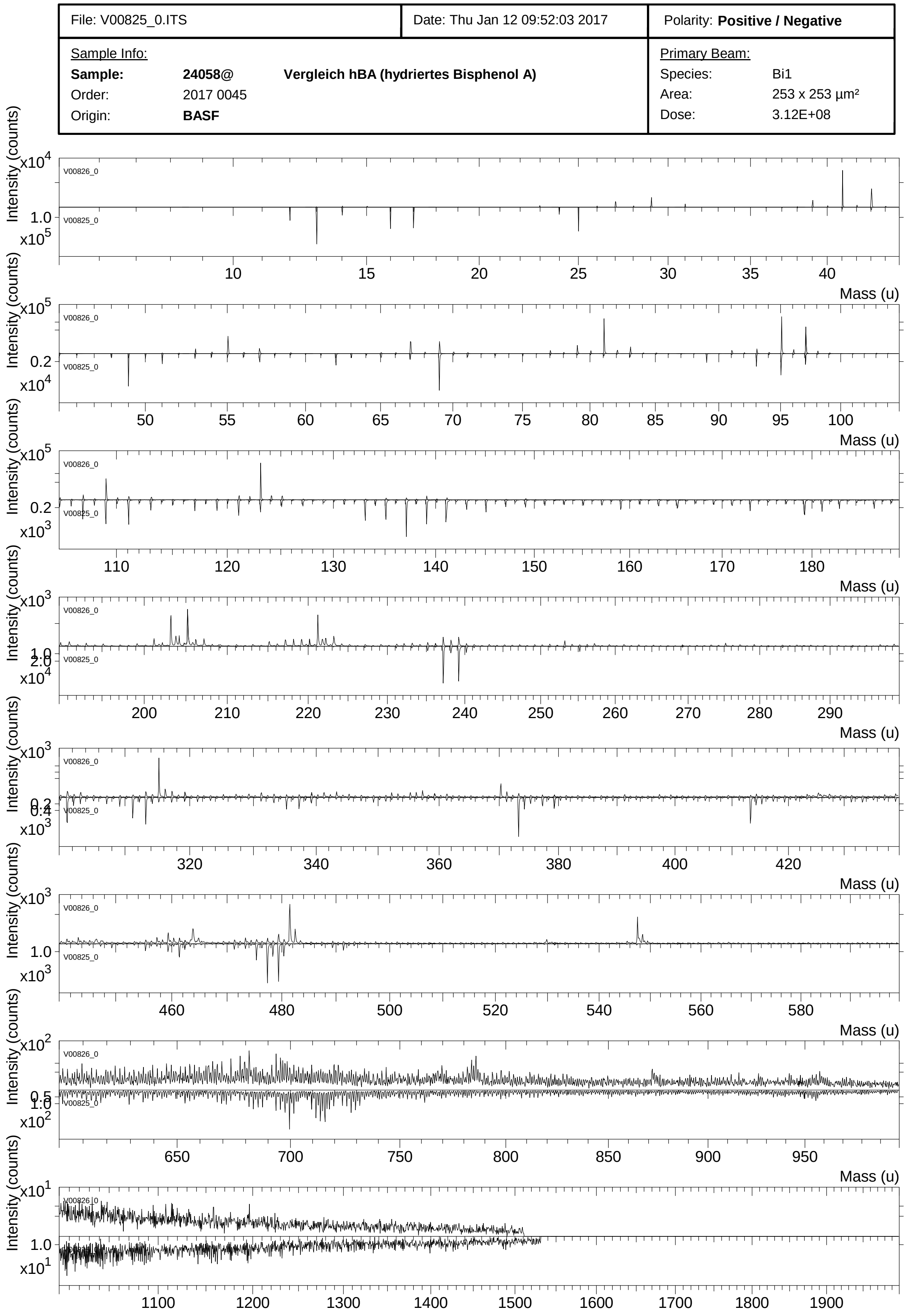

Figure S11 c. ToF-SIMS spectra of aqueous NaOH hydrolyzed 2-octenyl succinic anhydride (anhydride: MW = 210.3 g/mol, succinate: MW = 228.3 g/mol, disodium salt: 272.3 g/mol). Peaks of positive polarity are pointing upwards, those of negative polarity downwards. N.B.: real peak shape and fine structure (and thus precise mass) depend on representation resolution.

File: V01402_0.ITS			Date: Thu Feb 09 11:32:13 2017	Polarity: **Positive / Negative**	
Sample Info:				Primary Beam:	
**Sample:**	**24148@**	**n-Octenylbernstaeinsäure mit Na OH neitralisiert**		Species:	Bi1
Order:	2017 0045 B			Area:	136 x 136 µm²
Origin:	**BASF**			Dose:	3.12E+08

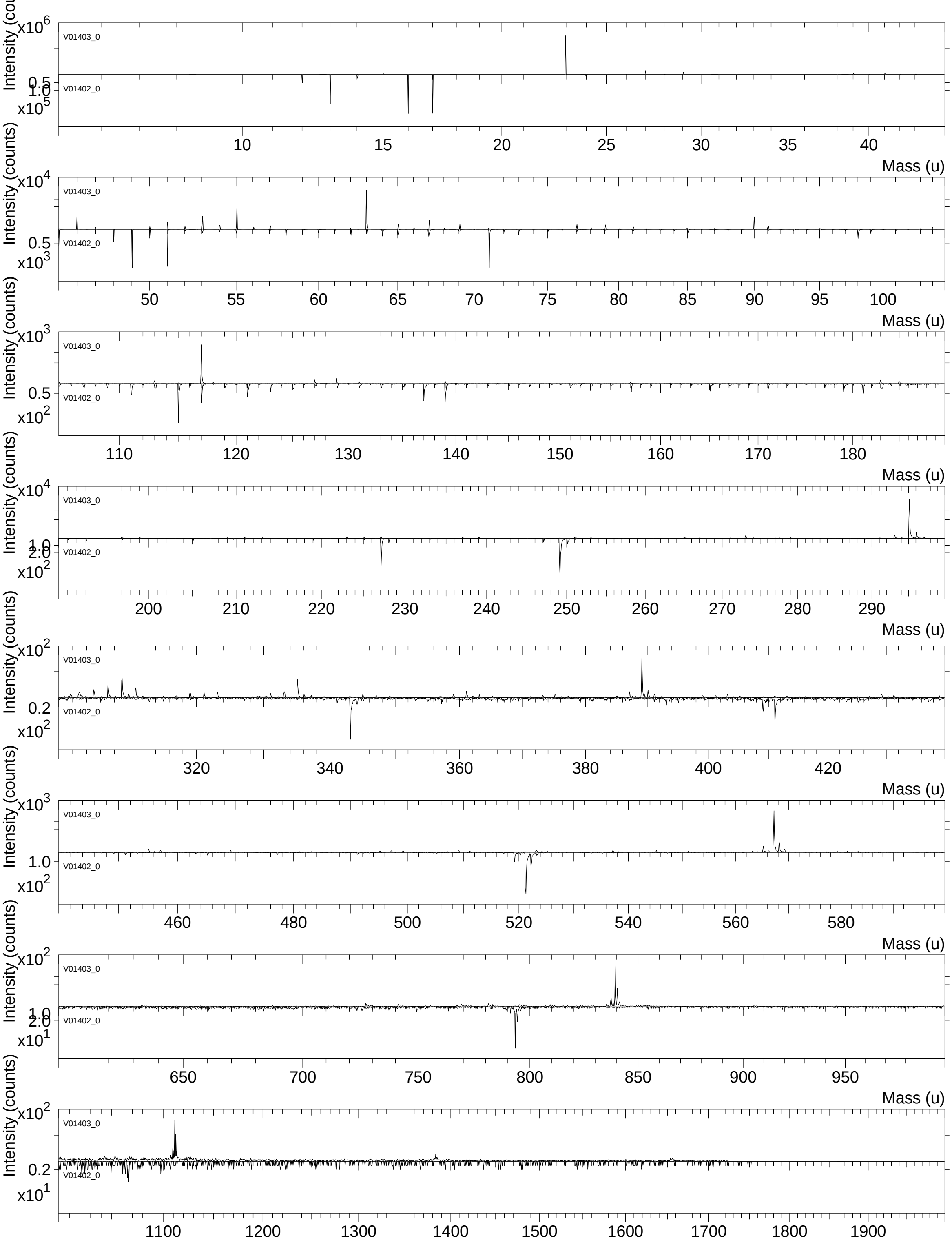

Figure S11 d. ToF-SIMS spectra of aqueous NaOH neutralized bola-amphiphile b-HBA (b-HBA: MW = 661 g/mol, disodium salt: MW = 705 g/mol). Peaks of positive polarity are pointing upwards, those of negative polarity downwards. N.B.: real peak shape and fine structure (and thus precise mass) depend on representation resolution.

File: V01400_0.ITS			Date: Thu Feb 09 11:19:56 2017	Polarity: **Positive / Negative**	
Sample Info:				Primary Beam:	
**Sample:**	**24147@**	**Bola-HBA mit NaOH neutralisiert**		Species:	Bi1
Order:	2017 0045 B			Area:	278 x 278 µm²
Origin:	**BASF**			Dose:	3.12E+08

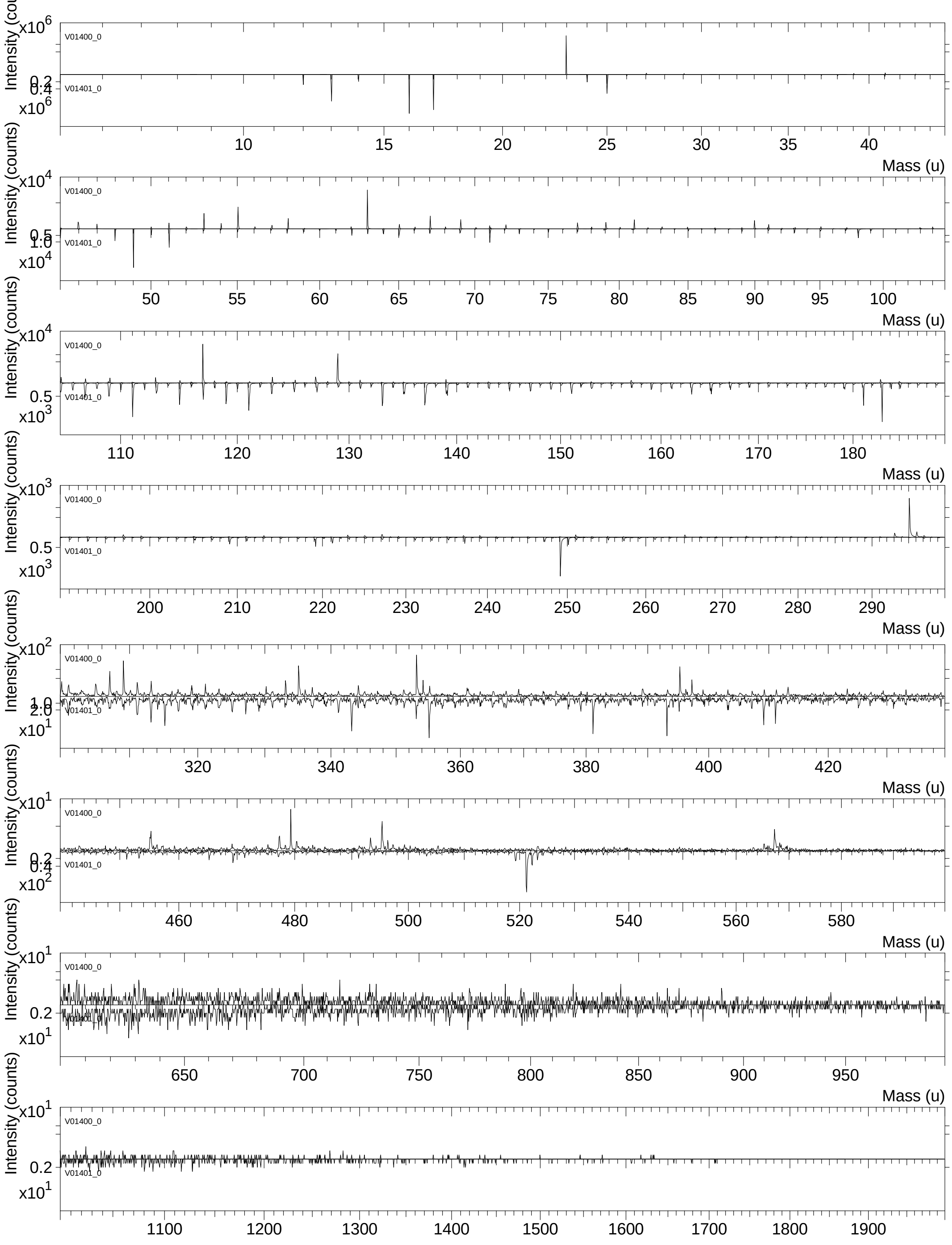

Figure S11 e. ToF-SIMS spectra (positive polarity) from the caustic soda treated LDH-NC sediment shell surface (upwards) and hydrogenated bisphenol-A (downwards). N.B.: real peak shape and fine structure (and thus precise mass) depend on representation resolution.

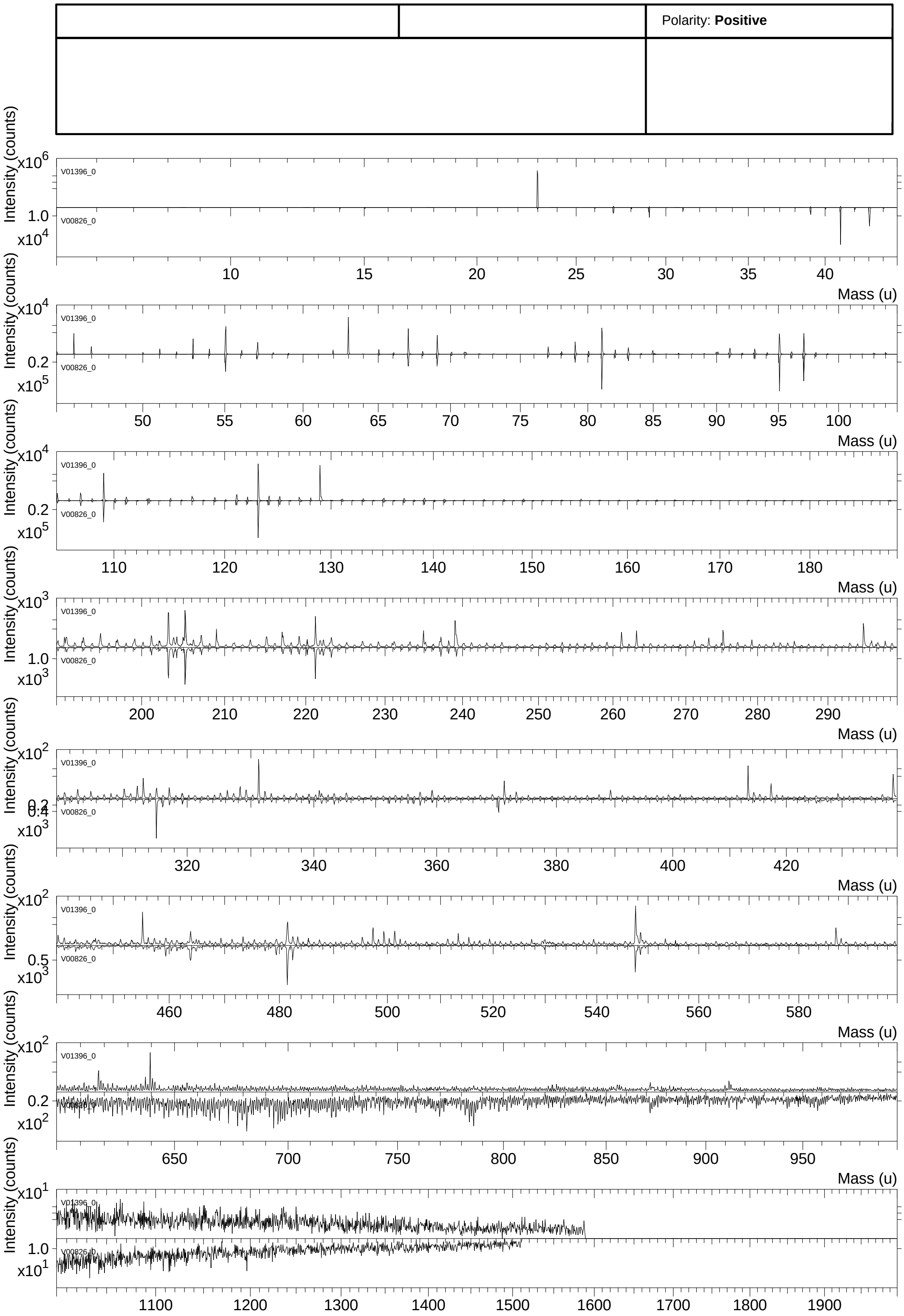

Figure S11 f. ToF-SIMS spectra (negative polarity) of the caustic soda treated LDH-NC sediment shell surface (upwards) and hydrogenated bisphenol-A (downwards). N.B.: real peak shape and fine structure (and thus precise mass) depend on representation resolution.

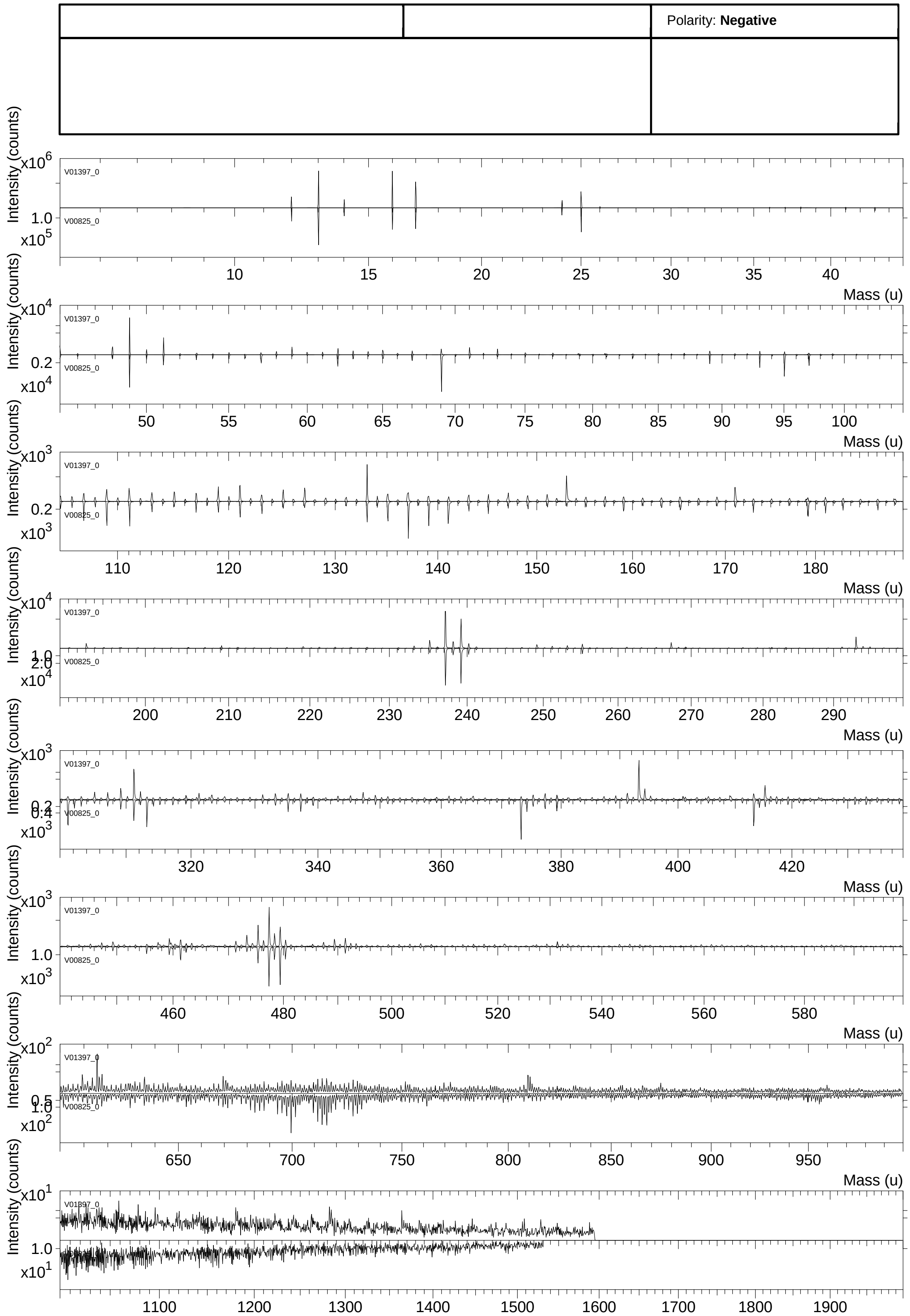

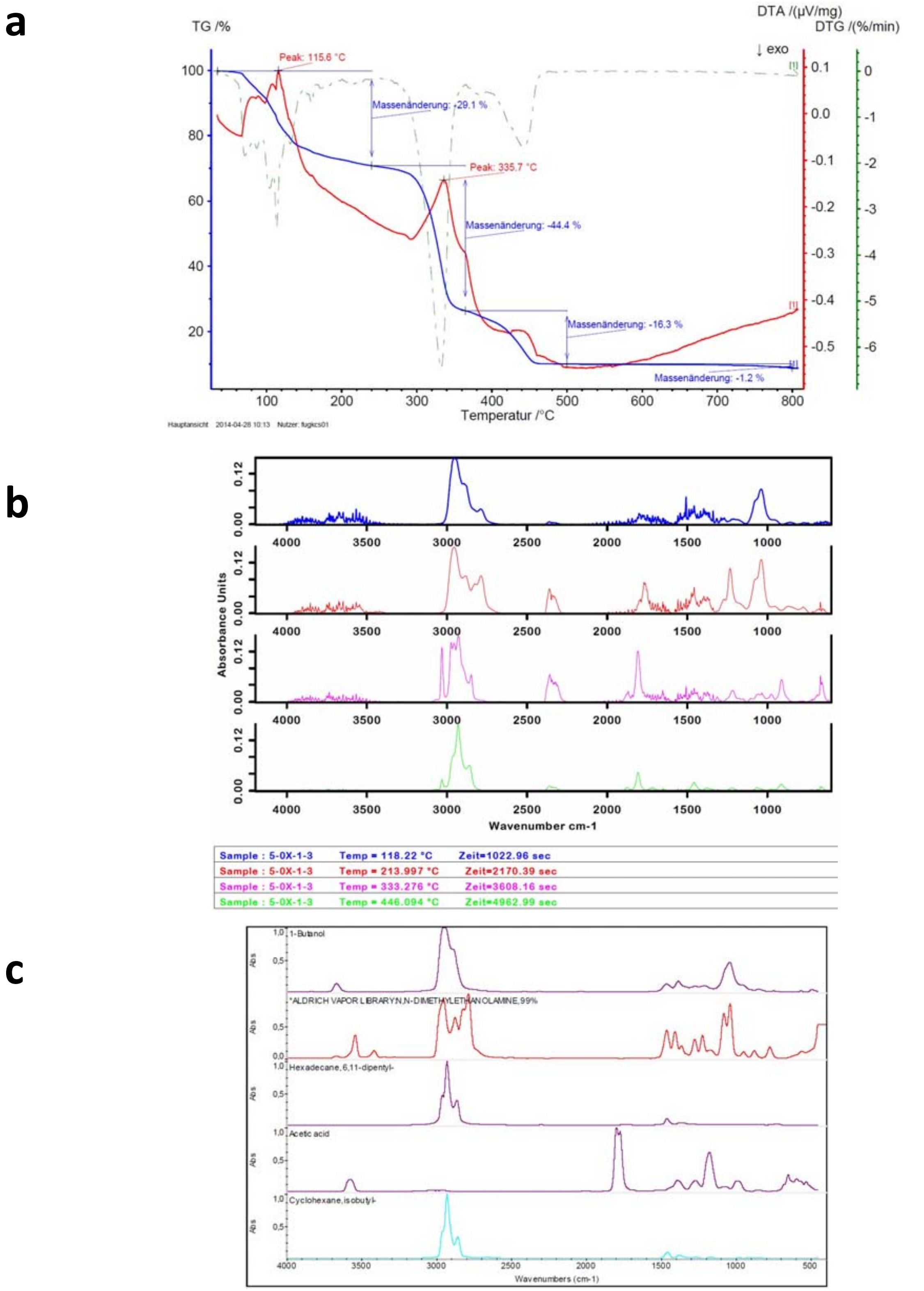

Figure S12. (a) TGA analysis of a physically dried (not cured) b-PES based LDH-BA sample (63.07 mg in Al_2O_3 crucible under Argon with 5 K/min). Heating from 35 to 140 °C nearly equals the residence time of a real coating in the oven (20 min). (b) Arbitrarily chosen FTIR spectra of the gaseous products, that were measured at 118 °C, 214 °C, 333 °C and 446 °C respectively. (c) Gas phase reference spectra of selected compounds.

Solvents, acetic acid and 2-(dimethylamino)ethanol substantially evaporate in the first step, although not quantitatively below 140°C. The sample was measured in a screening phase and differs from those in the article (here: w_{LDH} = 0.12 and n-butanol as solvent).

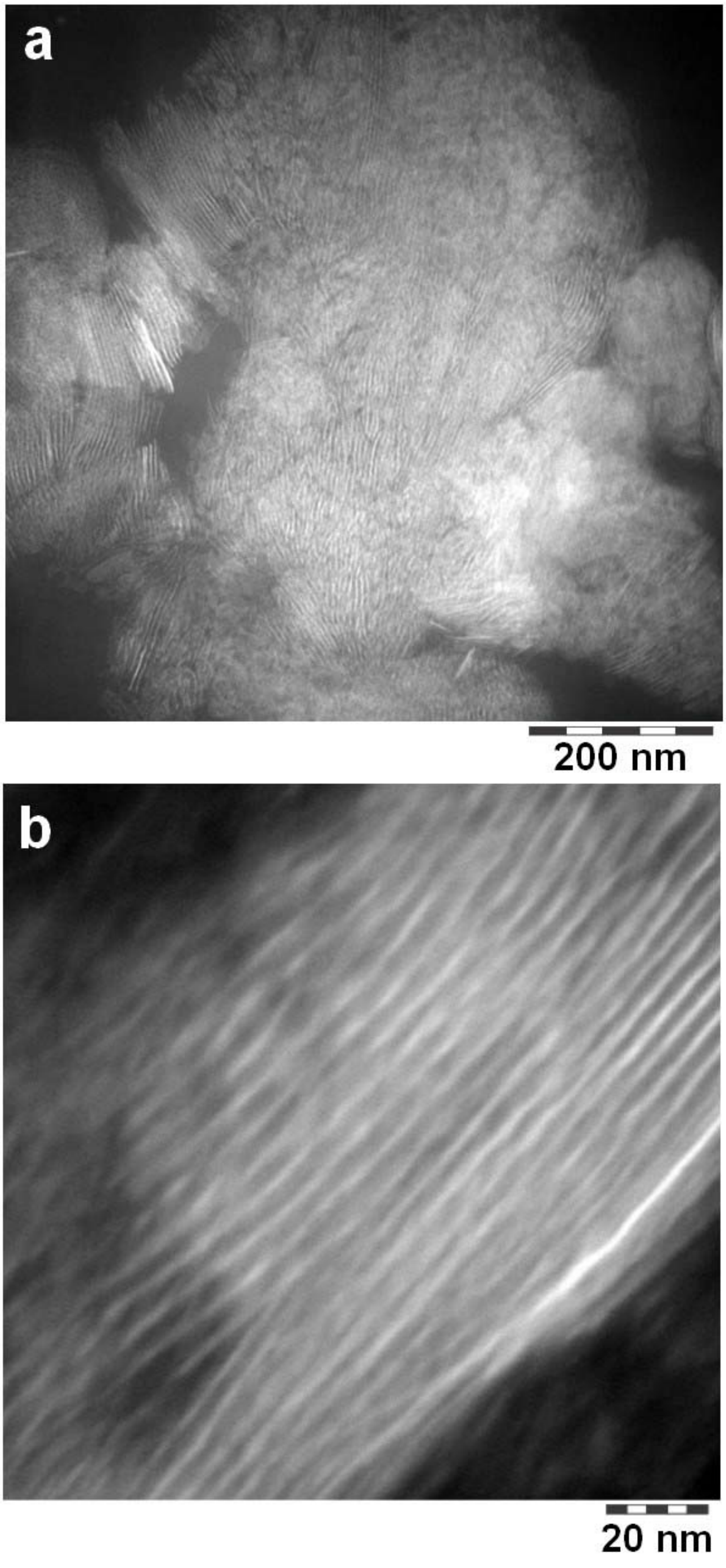

Figure S13. Cryo-TEM pictures of LDH-BA obtained from b-PES in n-propanol with charge ratio of 1:1 at ambient conditions.

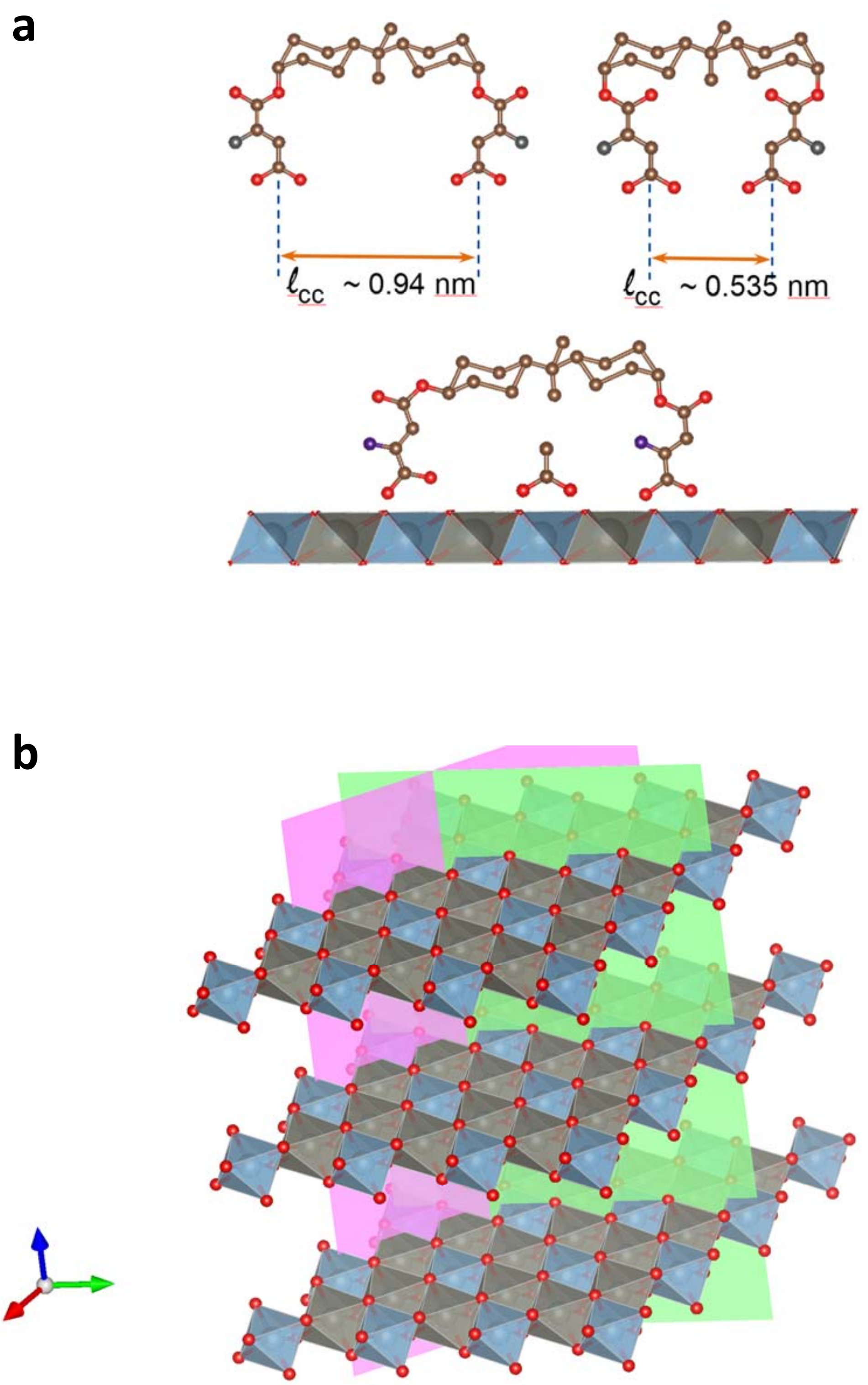

Figure S14. (a) Ball-stick models of cis, cis b-HBA (red: oxygen, purple: alkenyl side chain, only drawn in α position to ester group) and below a cis, trans b-HBA isomer that spans one Al^{3+} site with charge compensating acetate along the 100 lattice plane of the LDH that is shown in (b). The intra-layer distances between Al $^{3+}$ sites are 5.33 Å and 9.23 Å within the 100 and 110 lattice plane respectively. (b) Three stacked layers of the $Zn_2Al(OH)_6$ LDH framework of zincalstibite (intercalated $[Sb(OH)_6]^-$ and hydrogens omitted for clarity, grey octahedra comprise Zn^{2+}, blue ones Al^{3+}. The lattice plains 100 (mint) and 110 (pink) are highlighted. Structural data from: *Mineralogical Magazine*, 2012, **76**, 1337.

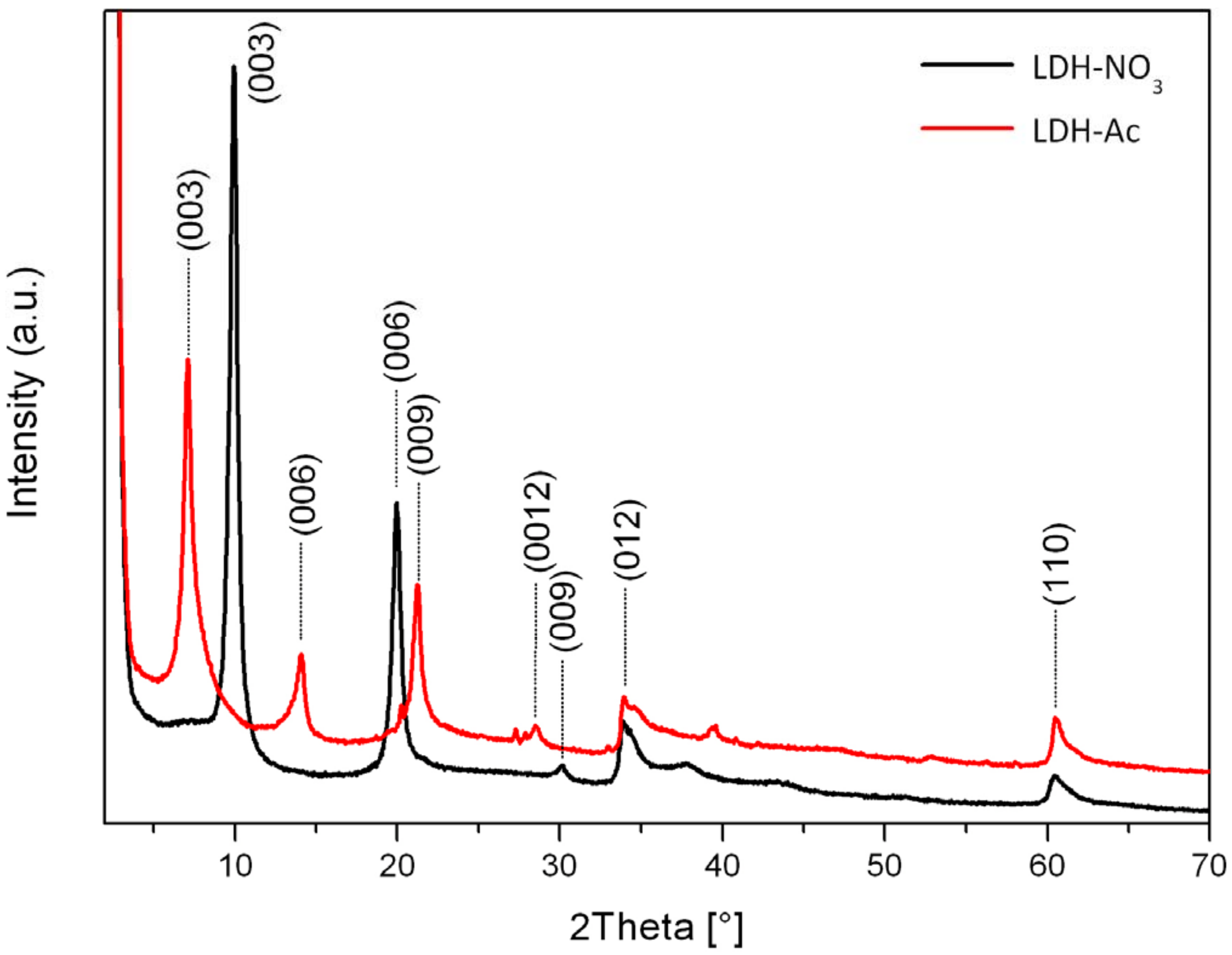

Figure S15. XRD diffraction curves of LDH-NO_3 phase (black) used for ion exchange against acetate and the product obtained (red, LDH-Ac). Diffraction peaks are allocated to the corresponding lattice plains. Peaks caused by (0kl) diffraction do not shift after anion exchange. Harmonic peaks for the layer stacking along the c axis (00l) are shifted to lower 2Θ values for LDH-Ac. Exchange of nitrate by acetate is associated with an increased repeat distance 1.244 nm versus 0.889 nm.

**Oxygen Permeation (23°C, 0% r.h.) ASTM D3985-05 (2010)**	Film Thickness [µm]		**Permeability** [cm³·µm/m²/day/bar]	
	mean	deviation	value	est. error
matrix (PUR / PES-NB / MF)	16.0	0.9	6.82E+04	7.07E+03
	18.1	1.8	7.37E+04	1.00E+04
matrix + b-PES	10.1	0.7	1.38E+05	2.39E+04
	10.8	0.5	7.50E+04	1.09E+04
matrix + PES-NB	14.1	0.7	9.66E+05	1.43E+05
	15.2	0.6	1.13E+05	1.60E+04
LDH-NC (b-PES)	12.0	0.4	3.81E+04	3.32E+03
	12.0	0.6	3.94E+04	5.88E+03
LDH-NC (PES-NB)	15.9	1.4	4.50E+04	8.34E+03
	14.8	0.4	4.34E+04	5.37E+03
**Oxygen Permeation (23°C, 85% r.h.) ASTM F 1927-07**	Film Thickness [µm]		**Permeability** [cm³·µm/m²/day/bar]	
	mean	deviation	value	est. error
matrix (PUR / PES-NB / MF)	16.0	0.9	7.28E+04	7.50E+03
	18.1	1.8	7.83E+04	1.17E+04
matrix + b-PES	10.1	0.7	1.96E+05	3.38E+04
	10.8	0.5	8.36E+04	1.21E+04
matrix + PES-NB	14.1	0.7	9.53E+05	1.41E+05
	15.2	0.6	1.21E+05	1.71E+04
LDH-NC (b-PES)	12.0	0.4	4.06E+04	3.54E+03
	12.0	0.6	4.21E+04	6.29E+03
LDH-NC (PES-NB)	15.9	1.4	4.71E+04	8.74E+03
	14.8	0.4	4.51E+04	5.58E+03

Table S1. Oxygen permeability measured on dried and humid free films, the liquid coatings being drawn with an applicator frame if not noted otherwise. (Abbreviations: see main text).

**Medium**	**n-propanol**				**water**			
**bola amphiphile**	**b-HBA**		**b-PES**		**b-HBA**		**b-PES**	
**charge ratio**	**1:1**	**5:1**	**1:1**	**5:1**	**1:1**	**5:1**	**1:1**	**5:1**
Ac- max theory [ppm]	896	703	766	466	947	621	883	644
Ac- found exp. [ppm]	30	27	34	17	42	12	65	59
Ac- recovered (%)	3.3	3.8	4.4	3.7	4.4	1.9	7.4	9.2
BA in film (s/s)	11.21	56.04	17.99	77.70	11.21	56.04	17.99	77.70
PUR, PES-NB, MF, P900 in film (s/s)	78.79	33.96	72.70	14.27	78.79	33.96	72.70	14.27
LDH in film (s/s)	10.00	10.00	9.31	8.04	10.00	10.00	9.31	8.04
mmol COOH / g film	0.46	1.09	0.56	1.41	0.46	1.09	0.56	1.41
µmol COOH	132	242	146	254	140	212	170	356

Table S2. Experimental data versus calculated data for acetate retrieval from baked and extracted films of LDH-NC. Calculated composition of solid films with values given for BA, LDH frame work and the film forming matrix composition (PUR, PES-NB, MF, P900). The losses of alcohols from MF-resin trans-etherification and 2-(Dimethylamino)ethanol are not considered.

	JCPDS 36-1451		LDH-BA		LDH-AM	
hkl	2 theta	I	2 theta	I	2 theta	I
**100**	31.76	57	31.83	100	31.83	100
**002**	34.42	44	34.47	19	34.47	47
**101**	36.25	100	36.31	51	36.31	89
**102**	47.53	23	47.61	11	47.61	27
**110**	56.60	32	56.70	30	56.70	38
**103**	62.86	29	62.92	8	62.92	20
**200**	66.37	4	66.46	10	66.46	15
**112**	67.96	23	67.99	10	67.99	21
**201**	69.09	11	69.16	12	69.16	18

Table S3. XRD diffraction peak positions and relative intensities for the Wurtzite ZnO structures of the samples shown in figure 6b of the article in comparison to data from the JCPDS card no. 36-1451. Measured intensities are not baseline corrected.
